# Alpha1a-Adrenoceptor Genetic Variant Triggers Vascular Smooth Muscle Cell Hyperproliferation and Agonist Induced Hypertrophy via EGFR Transactivation Pathway

**DOI:** 10.1371/journal.pone.0142787

**Published:** 2015-11-16

**Authors:** Irina Gradinaru, Ekaterina Babaeva, Debra A. Schwinn, Anush Oganesian

**Affiliations:** 1 Department of Anesthesiology & Pain Medicine, University of Washington, Seattle, Washington, United States of America; 2 Department of Anesthesiology, University of Iowa, Iowa City, Iowa, United States of America; 3 Department of Pharmacology, University of Iowa, Iowa City, Iowa, United States of America; 4 Department of Biochemistry, University of Iowa, Iowa City, Iowa, United States of America; Indian Institute of Technology Kanpur, INDIA

## Abstract

α_1a_ Adrenergic receptors (α_1a_ARs) are the predominant AR subtype in human vascular smooth muscle cells (SMCs). α_1a_ARs in resistance vessels are crucial in the control of blood pressure, yet the impact of naturally occurring human α_1a_AR genetic variants in cardiovascular disorders remains poorly understood. To this end, we present novel findings demonstrating that 3D cultures of vascular SMCs expressing human α_1a_AR-247R (247R) genetic variant demonstrate significantly increased SMC contractility compared with cells expressing the α_1a_AR-WT (WT) receptor. Stable expression of 247R genetic variant also triggers MMP/EGFR-transactivation dependent serum- and agonist-independent (constitutive) hyperproliferation and agonist-dependent hypertrophy of SMCs. Agonist stimulation reduces contractility Using pathway-specific inhibitors we determined that the observed hyperproliferation of 247R-expressing cells is triggered via β-arrestin1/Src/MMP-2/EGFR/ERK-dependent mechanism. MMP-2-specific siRNA inhibited 247R-triggered hyperproliferation indicating MMP-2 involvement in 247R-triggered hyperproliferation in SMCs. β-arrestin1-specific shRNA also inhibited 247R-triggered hyperproliferation but did not affect hypertrophy in 247R-expressing SMCs, indicating that agonist-dependent hypertrophy is independent of β-arrestin1. Our data reveal that in different cardiovascular cells the same human receptor genetic variant can activate alternative modulators of the same signaling pathway. Thus, our findings in SMCs demonstrate that depending on the type of cells expressing the same receptor (or receptor variant), different target-specific inhibitors could be used to modulate aberrant hyperproliferative or hypertrophic pathways in order to restore normal phenotype.

## Introduction

Adrenergic receptors (ARs) are activated by the sympathetic nervous system catecholamines norepinephrine and epinephrine and play a major role in regulating cardiovascular function during physiological and/or pathological conditions. Elevated levels and prolonged effects of plasma catecholamines are risk factors for development of vascular diseases [1,2], such as vessel wall hypertrophy, atherosclerosis, and restenosis after vessel injury. Direct stimulation of α_1_-adrenergic receptors (α_1_ARs), members of G protein-coupled receptor (GPCR) superfamily, has been shown to induce dose-dependent proliferation, hypertrophy, and migration of vascular smooth muscle cells (SMCs) and adventitial fibroblasts [3–8]. In injured arteries, the potency of these effects is highly increased [7].

α_1_ARs are also important regulators of vascular tone and play a major role in blood vessel repair. Numerous studies demonstrate that activation of α_1_ARs leads to vasoconstriction, and knockout of these receptors in mice results in impaired maintenance of normal arterial blood pressure (BP) [9]. Three subtypes of human α_1_AR (α_1a_, α_1b_, α_1d_) have been cloned and pharmacologically characterized. Although precise physiological rationale for having three α_1_AR subtypes remains elusive, differential subtype function is supported by differences in tissue distribution, G-protein-coupling, and response to agonist stimulation [10,11]. Vascular α_1_ARs have been extensively studied in animal models and it has been suggested that all three subtypes play a role in BP control [9,12–14], although the α_1_AR subtype-specific contraction differs from the animal models used or vascular bed investigated [15].

Human vascular α_1_AR subtype distribution is distinct from other animal models: α_1_AR subtypes vary with vessel bed, correlate with contraction in mammary artery and vary with age [16]. α_1a_ARs are major subtype in human heart and vascular SMCs, particularly in resistance vessels and are involved in BP control regardless of age. However, concurrent vascular α_1b_ expression is increased in older individuals (>65 years), with both subtypes ultimately involved in BP maintenance [16]. In terms of signal transduction, stimulation of all three α_1_AR subtypes with receptor agonist results in activation of the G_q/11_ signaling pathway, including activation of phospholipase C, generation of second messengers inositol (1,4,5) triphosphate and diacylglycerol, and mobilization of intracellular calcium. Although all three α_1_AR subtypes activate the same G_q/11_ protein signaling pathway, different human tissue distributions suggest they may play distinct functional roles. While the canonical, mitogenic signaling pathways activated by GPCRs in general (and by α_1_ARs in particular) are reasonably well defined [17], the less studied, but equally important is GPCR signaling through appropriate receptor tyrosine kinases such as the epidermal growth factor receptor (EGFR). To date, most studies of EGFR transactivation by vasoactive GPCRs have centered on the growth effects of these GPCRs and their potential impact on development of cardiovascular hypertrophy upon agonist stimulation [18–20]. However, it is conceivable that EGFR transactivation modulates vascular tone as well as growth [21,22]. It has been demonstrated that catecholamine-induced activation of α_1_ARs triggers protein synthesis in rat medial aortic SMCs, which is a process mediated by the ROS-dependent EGFR/ERK transactivation pathway [23]. Also, contractions of rat thoracic aorta by α_1_ARs involving EGFR transactivation pathway and mediated via PI3K and ERK1/2 activation has been demonstrated in another study [24]. However, only very few studies presenting EGFR transactivation via agonist-dependent α_1_ARs activation describe which particular α_1_AR subtype is responsible for the pathway activation. Hao et al. demonstrated that stimulation of α_1b_-adrenoceptors in arteries results in activation of matrix metalloproteinase 7 (MMP-7), and subsequent EGFR-dependent signaling contributing to the vascular tone [25]. It has been reported that in GT1-7 neuronal cells EGFR transactivation by α_1a_AR involving HB-EGF release requires both PKC and Src activation [26], however, in these cells again proliferation is more associated with α_1b_AR subtype [27]. α_1a_ARs play a role in both SMC and fibroblast proliferation during vessel injury [7,28]. We have demonstrated that upon agonist stimulation α_1a_ subtype triggers small, but statistically significant EGFR transactivation-dependent proliferation in Rat-1 fibroblasts when receptor expression is close to physiological levels [29] or during very small window of low agonist concentration [30].

In addition, GPCRs can activate EGFR through G_q_ and/or β-arrestin-dependent pathways. Recent advances in GPCR research illustrate the importance of β-arrestins in regulating discrete signaling pathways with unique biological outcomes [31]. Agonist-mediated, β-arrestin- or G protein-dependent EGFR transactivation has been demonstrated for β_1_- and β_2_ARs [32,33] as well as for angiotensin receptor AT1a [34]. Different physiological responses of GPCRs observed by G protein- or β-arrestin-biased signaling is a promising new paradigm in cardiovascular research leading to the possibility of new approaches and selective therapies [35].

Polymorphisms in genes encoding sympathetic ARs, including α_1a_AR SNPs, are also associated with hypertensive disorders and heart failure [36–39]. Nine naturally occurring human AR SNPs identified in the coding region and affecting different domains of α_1a_AR have been characterized pharmacologically [40]. Among these, the G247R SNP (247R), located in the third intracellular loop (3iL) of the α_1a_AR receptor is particularly interesting since it was originally identified in a patient with severe hypertension (HTN). The 3iL is functionally important for G_q_ protein coupling and intracellular signaling and is not directly involved in ligand binding.

Recently, our laboratory discovered a novel signaling mechanism triggered by the naturally occurring α_1a_AR-247R (247R) genetic variant. We reported that 247R expression results in hyperproliferation of rat fibroblasts due to MMP-7/ADAM-12-mediated, constitutively active (agonist-independent) coupling to EGFR transactivation pathway that is G_q_-independent but β-arrestin1-dependent. Agonist treatment of wild type receptor triggers only small, but statistically significant, EGFR-dependent cell proliferation in fibroblasts [29]. Our recent findings also reveal that EGFR transactivation by 247R triggers agonist-independent hyperproliferation and agonist-dependent hypertrophy in cardiomyoblasts [41], suggesting a common mechanism that is not cell type-dependent.

Since vascular SMCs are the most clinically relevant model, particularly given α_1a_AR’s role in human vessels, in the present study we used rat aortic and human coronary artery vascular SMCs. Specifically, we tested the hypothesis that constitutive expression of α_1a_AR-247R genetic variant in SMCs, triggers MMP- and EGFR-dependent cell proliferation and/or hypertrophy, and if this pathway is β-arrestin-dependent. We also tested whether the pathway-specific small molecule inhibitors would be effective in restoring the normal phenotype, and whether activation of ERK in rat vascular SMCs is mediated via β-arrestin signaling. Our results not only demonstrate the importance of β-arrestin-mediated signaling in ERK-dependent proliferative responses of SMCs, they also shed new light on molecular mechanisms and interrelationships between the β-arrestin and classical G protein-mediated activation of these pathways. A final novel finding in this study is that 247R cells exhibit higher proliferative capacity in three dimensional (3D) cultures in collagen gels and demonstrate statistically significant increased contractile function under the basal conditions compared with WT control.

## Materials and Methods

### Cell culture

A-10 embryonic rat aorta thoracic/medial layer-derived SMCs (ATCC^®^ CRL-1476, Manassas, VA) were cultured in DMEM (Gibco, Auckland, NZ) supplemented with 10% FBS (Hyclone Laboratories, South Logan, UT) and 100U/ml penicillin/streptomycin (Gibco, Grand Island, NY) at 37°C in 5% CO_2_. Cells were subcultivated every 4 days, and experiments were performed in 0%, 0.5%, or 10% FBS-containing medium as indicated. Human coronary artery smooth muscle cells (Life Technologies, C-017-5C, Carlsbad, CA) were cultured in Medium 231 supplemented with Smooth Muscle Growth Supplement (SMGS, Life Technologies,) at 37°C in 5% CO_2_. Cells were subcultivated every 5 days or at 80% confluency and experiments were performed in 0.5% SMGS-supplemented medium.

### Cell lines

To establish A-10 SMCs stably expressing HA-α_1a_AR-WT or HA-α_1a_AR-247R, cells were transfected with pcDNA3 plasmid containing human hemagglutinin epitope (HA) tagged α_1a_AR-WT (WT) or α_1a_AR-247R (247R) genetic variant using Neon Transfection System (Life Technologies). 150,000 cells and 1.25μg of plasmid DNA were used for each transfection in a 6-well plate using electroporation (1400V, 20ms and 2 pulses) followed by plating the cells in 10% FBS-containing DMEM without antibiotics and culturing for 48h. Transfection efficiency was determined by enhanced green florescence protein (EGFP) expression. Cells were selected based on resistance to G418 (800μg/ml for 2 weeks) and individual clones were isolated and expanded in 400μg/ml G418-containing medium. Receptor expression was determined by radioligand-binding assays using [^125^I]-HEAT (Perkin Elmer, Boston, MA) as described in [29] and clones with low and comparable receptor levels (<300fmol/mg protein) were used in subsequent experiments.

### Transient transfection

For human coronary artery SMCs, experiments were performed in transiently transfected cells expressing WT or 247R receptors. Briefly, cells were transfected with pcDNA3 plasmid containing HA-tagged α_1a_AR-WT or HA-tagged α_1a_AR-247R using Neon Transfection System. 1.5x10^6^ cells and 6μg of plasmid DNA were used for each transfection. The parameters for electroporation were set to 1400V, 20ms and pulse of 1. These transfection parameters resulted in cells expressing ~300fmol/mg receptor, as determined by radioligand-binding assays using [^125^I]-HEAT.

### Cell proliferation

Cells were plated in 12-well plates at a density of 20,000 cells per well for experiments in 0.5% FBS-containing medium (or 15,000 cells per well for 10% FBS-containing medium) in the presence or absence of agonist: 10μM phenylephrine (PE), or 100nM A61603 Hydrate {N-[5-(4,5-dihydro-1H-imidazol-2yl)-2-hydroxy-5,6,7,8-tetrahydronaphthalen-1-yl] Methanesulphonamide hydrobromide}, (Sigma-Aldrich, St. Louis, MO) or various inhibitors at the following final concentrations: 10μM PD98059 (Cell Signaling, Danvers, MA or Enzo Life Sciences, Farmingdale, NY), 5μM UO126, 500nM or 1μM AG1478 (Cell Signaling), 10 or 25μM GM6001 and 1.5μM PP2 (Calbiochem, San Diego, CA). To block β-adrenergic receptors, 10μM propranolol (Sigma-Aldrich) was added 30min before adding agonists or inhibitors and the cells were cultured for 48h. Proliferation experiments in human coronary artery SMCs were performed in 12-well plates at a density of 50,000 cells per well and grown in 0.5% SMGS-supplemented medium with or without agonist (10μM PE or 100nM A61603 Hydrate) stimulation for 24 or 48h. In the proliferation assays where α_1a_AR inverse agonist prazosin was used, A-10 cells stably expressing vector only, WT or 247R were plated in 12-well plates at a density of 20,000 cells per well in DMEM supplemented with 10% FBS. The next day medium was changed to DMEM supplemented with 0.5% FBS and 10μM propranolol with or without 1μM prazosin and the cells were cultured for an additional 48h. For all cell proliferation experiments, at least 3 independent experiments plated in triplicates were performed and cell numbers were determined by light microscopy.

### β-Arrestin1 knockdown with shRNA

Control A-10, and A-10 cells stably expressing HA-α_1a_AR-WT or HA-α_1a_AR-247R receptors were plated into 6-well plates or 35-mm glass-bottom plates and transfected with 4μg β-arrestin1-specific or scrambled shRNA using Lipofectamine 2000 as previously described [29]. Transfection efficiency was ~60% as determined by fluorescent imaging of EGFP-transfected cells. The following day cells from 6-well plates were trypsinized, counted, and replated onto 12-well plates at 25,000 cells per well in triplicates for the proliferation assays or onto gelatin-coated 35mm glass-bottom plates at 15,000 cells per plate to label with fluorescein-conjugated wheat germ agglutinin for evaluation of cell surface area as described below. Cells were grown for 24h in DMEM supplemented with 0.5% or 10% FBS, 10μM propranolol and with or without 10μM PE. In proliferation assays the resultant cell numbers were enumerated by light microscopy.

### Immunoblotting

For Western blot analyses, cells were cultured in 0.5% FBS-containing medium with 10μM propranolol and treated with or without EGFR-specific inhibitor AG1478 (1μM) or MMP inhibitor GM6001 (25μM) in the presence or absence of 10μM PE for 48h. DMSO was used as a control vehicle. Cells were lysed in lysis buffer (50mM Tris, pH 7.4, 150mM NaCl, 15mM NaF, 7.5mM Na_3_VO_4_, 3.75mM β-glycerolphosphate, 1% Triton X-100) containing protease inhibitor cocktail (Roche, Indianapolis, IN or Pierce, Rockford, IL). Approximately 30–70μg of total protein was separated on SDS-polyacrylamide gel, followed by electroblotting onto a nitrocellulose membrane and probing with specific antibodies. After immunoblotting, membranes were incubated with secondary antibodies conjugated with peroxidase and protein bands were visualized with ECL substrate (Perkin Elmer, Pierce, or Millipore, Temecula, CA). Membranes were stripped and reprobed with anti-actin antibody to confirm equal protein loading. Densitometric analysis was carried out using Adobe Photoshop Elements 10 (Adobe, San Jose, CA). The following antibodies were used: phospho-p44/42 MAPK (Erk1/2) (Th202/Tyr204) (D.13.14.4E), XP rabbit mAb; p44/42 MAPK (Erk1/2) rabbit polyclonal; phospho-Akt (Ser473) rabbit polyclonal; Akt rabbit polyclonal all from Cell Signaling Technology, 4G10 Platinum Anti-Phosphotyrosine (pY) mouse monoclonal (Millipore); Anti-Epidermal Growth Factor Receptor [pY^845^] Phophospecific, unconjugated rabbit polyclonal (Life Technologies); anti-β-actin antibody [AC-15] mouse monoclonal (Abcam, Cambridge, MA).

### MMP-2 knockdown with siRNA

Control A-10 cells stably expressing vector plasmid, WT or 247R receptors were plated into 6-well plates and transfected with 50nM non-targeting negative control (4390843, Life Technologies) or silencer predesigned siRNA against MMP-2 (s135546, Life Technologies) using Lipofectamine 2000 as previously described [42]. The following day, cells were trypsinized, counted, and re-plated onto 12-well plates at 30,000 cells per well in triplicates for evaluation of cell proliferation. Cells were grown for 24h in DMEM supplemented with 0.5% or 10% FBS. The resultant cell numbers were enumerated by light microscopy. siRNA specificity was analyzed by RT-PCR as described below.

### RNA isolation and RT-PCR analysis

RNA was isolated from A-10 control, WT or 247R-expressing cells using RNeasy Plus Kit (Qiagen, Germantown, MD) according to manufacturer’s instructions. cDNA was generated using 200ng RNA (in MMP-2 siRNA experiments) or 3μg RNA and RevertAid First Strand cDNA Sythesis Kit (Thermo Scientific, Waltham, MA). Gene-specific primers used for MMP-2, MMP-7, MMP-9, ADAM-10, ADAM-12, ADAM-17, and GAPDH as an internal control were:

MMP-2 (sense) 5′-GTATGGAGCGACGTAACTCCAC-3’,

(anti-sense) 5′-GCAGCTCTCATACTTGTTGCC-3’;

MMP-7 (sense) 5'-GTGTCATGG AGATAATGCAGAA-3',

(anti-sense) 5'-GAATGATCTCCTTGATAGGTAGG-3';

MMP-9 (sense) 5′-AGTTTGGTGTCGCGGAGCAC-3′,

(anti-sense) 5′-TACATGAGCGCTTCCGGCAC-3′;

ADAM-10: (sense) 5′-CCAGCAGAGAGATATATTAAAGAC-3’,

(anti-sense) 5′-ATCCAAAGTTTATGTCCAACTTCA-3’;

ADAM-12: (sense) 5′-ATGACCAAGTATGTAGAACTGGTTA-3′,

(anti-sense) 5′-AGAATTCTGGGAGGTCGCAGGAGTTG-3′;

ADAM-17: (sense) 5′-GAATATAACATAGAGCCACTTTGGA-3′,

(anti-sense) 5′-CTTTGTAAGGATGGTTTTACCATA-3′;

GAPDH: (sense) 5’-GAAGGGCTCETGACCACAGTCCAT-3’,

(anti-sense) 5’-TAGCCATATTCGTTGTCGTTGTCGTACCAGG-3’. PCR amplifications were performed using GoTaq Green Master Mix (Promega, Madison, WI) according to the manufacturer’s recommendations. PCR conditions for MMP-2 were 95°C 30s, 60°C 30s, and 72°C 30s, for a total of 30 cycles; for MMP-7: 95°C 30s, 58°C 30s, and 72°C 30s, for a total of 35 cycles; for MMP-9 (30 cycles), for all ADAMS and GAPDH (25 cycles): 95°C 30s, 55°C 30s, and 72°C 30s. After the last cycle the samples were incubated for an additional 10min at 72°C. Gel images were acquired using Gel Doc LT Imaging Systems (BioImaging Systems, Upland, CA).

### Gelatin zymography for MMP-2

Cells were cultured in serum-free DMEM for 24h. Media was collected and concentrated at 4°C by ultrafiltration using Amicon Ultra columns (Millipore). Media equivalent to 30x10^3^ cells was run on 10% gelatin zymography gels (Bio-Rad Laboratories, Hercules, CA). Gels were washed in 2.5% Triton X-100 three times 10min each and incubated overnight in developing buffer (50 mM Tris pH 8.2, 5 mM CaCl_2_, 0.5 μM ZnCl_2_) at 37°C, followed by staining with Coomassie brilliant blue and destaining. Gel images were acquired using Scan Maker i900 (Microtek, Industrial Park Hsinchu, Taiwan).

### Evaluation of 3D collagen gel contraction

Rat tail collagen (kindly provided by Dr. Zhang, University of Washington) was diluted in DMEM supplemented with 10% FBS and NaOH to achieve a final concentration of 3mg/ml collagen in DMEM at pH 7.4. Cells were trypsinized, pelleted and resuspended in collagen solution at 10^6^ cells/ml. The collagen-cell suspension (100 μl) was pipetted into the wells of 96-well flat-bottom plates and incubated for 20min at 37°C, followed by addition of 10% FBS-containing DMEM. The next day, new medium with or without 10μM PE was added and the plate was incubated for additional four days with daily refreshment of the medium and PE. Gels were fixed in PBS solution of 3.7% formaldehyde for 20min at room temperature, the gel images were acquired using Nikon microscope and the gel sizes were evaluated using NIS-Elements AR software (Nikon Instruments Inc, Melville, NY). Subsequently, the gels were digested with 20mg/mL collagenase type 2 (Worthington Biochemical Corporation, Lakewood, NJ) in PBS supplemented with 5mM CaCl_2_ at 65°C and the cell numbers were determined using light microscopy. Cell contractility is presented as percent of gel area reduction normalized to 1000 cells as compared to non-stimulated control cells.

### Evaluation of cell surface area

Cells were plated onto 35mm glass-bottom plates and next day medium was changed to 0.5% or 10% FBS-containing medium with 10μM propranolol and cells were cultured for an additional 48h with or without 10μM PE. In the experiments with α_1_AR inverse agonist prazosin, cells were cultured in the presence or absence of 1μM prazosin or/and 1 μM PE. Cell membranes were labeled with fluorescein-conjugated wheat germ agglutinin (Alexa Fluor^®^ 488 conjugate, Life Technologies) [43] for 5min, washed twice with DMEM and then with PBS. Subsequently, cells were fixed in 10% neutral buffered formalin solution for 10min at room temperature. To visualize nuclei, cells were covered with mounting medium containing 4',6-diamidino-2-phenylindole (DAPI Mounting Medium, Vector Laboratories, Burlingame, CA). Images were captured using Nikon DS-Ri1 Microscope (Nikon Instruments Inc.) with a 20x objective. For each independent experiment, we evaluated a minimum of 5 fields per cell type using Image J software. When cells were transfected with EGFP-β-arrestin-specific or scrambled shRNA followed by PE treatment only EGFP-positive cell surface area was evaluated for each cell type.

### Statistical analysis

Statistical comparisons were made with one- or two-way analysis of variance (ANOVA) using GraphPad Prism (GraphPad Software, La Jolla, CA) followed by post-hoc Tukey's or Bonferroni test as indicated. Statistically significant differences are indicated by *p<0.05, **p<0.01, ***p<0.001.

## Results

### HA-α_1a_AR-247R (247R) expression in SMCs triggers constitutive cell proliferation that is inhibited by agonist stimulation

**Fig 1:**
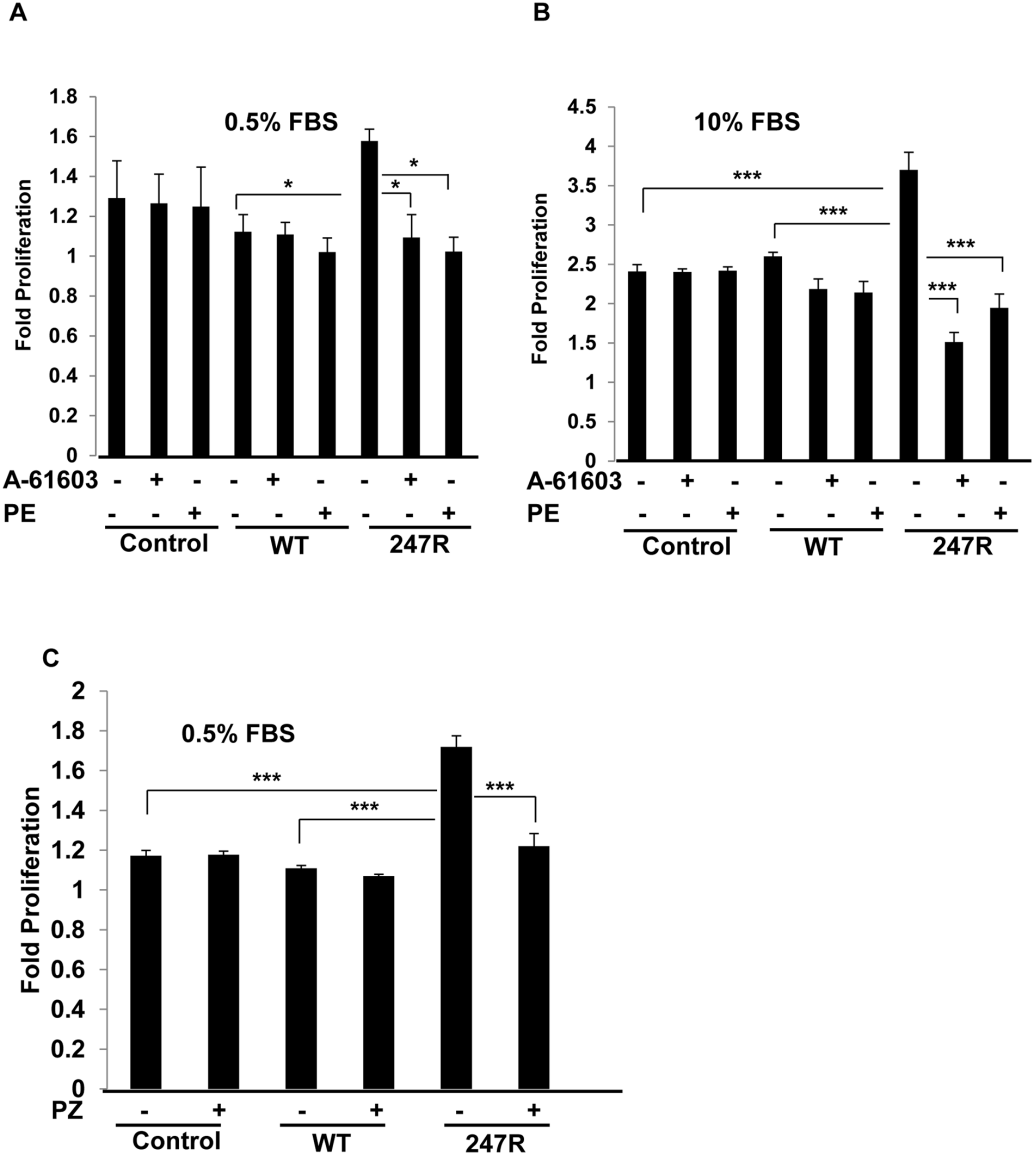
247R expression in SMCs triggers constitutive hyperproliferation. Agonist treatment inhibits increased proliferation. A-10 SMCs stably expressing control plasmid, WT or 247R receptors were cultured in (A) 0.5% FBS or (B) 10% FBS-containing medium for 48h in the presence or absence of agonist (A61603 or PE), or (C) in the presence or absence of 1μM prazosin (α_1_AR inverse agonist). Cell proliferation was evaluated by cell counting using light microscopy. Data represent mean ± SE of 3 independent experiments each performed in triplicates and analyzed by two-way ANOVA followed by post-hoc Tukey’s test.

To determine whether 247R-triggered hyperproliferation in cardiovascular cells [29,41] is not cell type dependent but is a generalizable phenomenon, we examined proliferation of SMCs stably expressing HA-α_1a_AR-247R (247R), HA-α_1a_AR-WT (WT) or empty vector (control) in the presence or absence of agonists. To be close to expression levels of endogenous receptors, we purposefully used clone pairs of similar receptor densities of 247R and WT, approximately 300 fmol/mg protein, as determined by radioligand binding assays. Cell proliferation studies reveal that A-10 SMCs stably expressing 247R exhibit ~1.5–2 fold increased constitutive hyperproliferation compared to WT or control cells when cultured in the presence of low 0.5% or 10% serum conditions (Fig 1A and 1B). Transient expression of 247R in primary human coronary artery SMCs also results in ~20% increased proliferation compared to control cells or cells expressing WT receptors (S1 Fig). Treatment with agonists (10μM PE or 100nM A61603) inhibits the enhanced proliferation rate of 247R cells in A-10 SMCs or human coronary artery SMCs compared to that of WT or control cells (Fig 1A and 1B and S1 Fig).

To determine if increased proliferation of 247R-expressing cells is a result of receptor constitutive activity, cells were treated with 1μM prazosin, a known α_1_AR inverse agonist, and baseline cell proliferation was evaluated. As demonstrated in Fig 1C, prazosin treatment inhibits 247R-mediated cell proliferation while having no effect on WT or control cell proliferation. These data demonstrate that 247R-triggered SMC proliferation is a receptor constitutive activity-dependent.

### Increased proliferation of 247R-expressing cells is Src-, MMP- and EGFR-dependent

**Fig 2:**
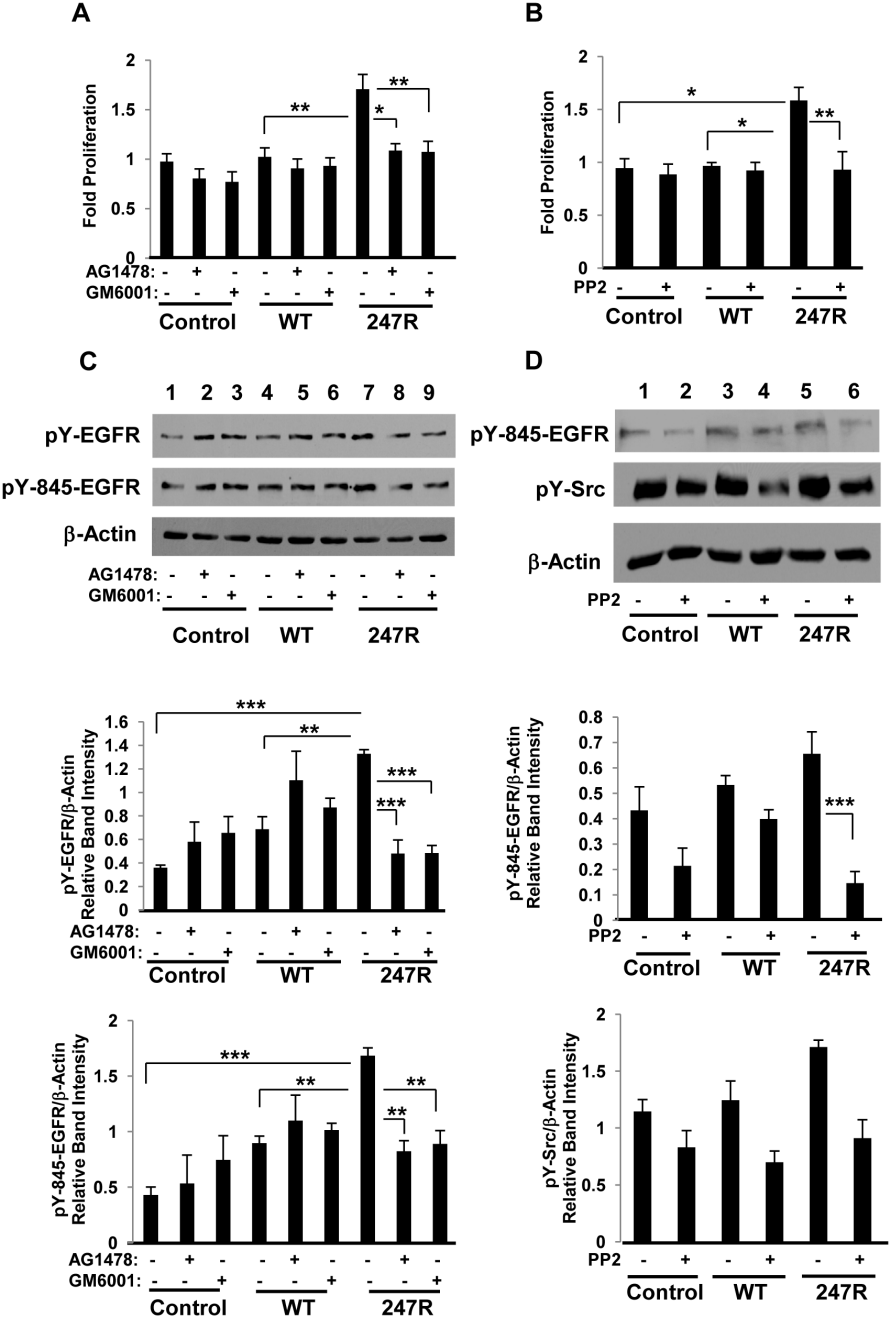
247R expression triggers Src/MMP-dependent EGFR transactivation. (A,C) A-10 cells stably expressing control plasmid, WT or 247R receptors were cultured in the presence or absence of EGFR-specific inhibitor AG1478, MMP inhibitor GM6001 for 48h, or (B, D) Src-specific inhibitor PP2 for 24h in 0.5% FBS-containing medium. Cell proliferation was evaluated by cell counting using light microscopy and cell lysates were analyzed by Western blotting. Lower panels depict relative intensities of phospho-protein bands as determined by densitometric analysis. Data represent mean ± SE of 3 independent experiments and analyzed by two-way ANOVA followed by post-hoc Tukey’s test.

We have previously demonstrated that expression of HA-α_1a_AR-247R (247R) receptor in fibroblasts and cardiomyoblasts triggers EGFR-transactivation-dependent constitutive hyperproliferation [29,41]. To determine whether the constitutive hyperproliferation of SMCs stably expressing 247R receptor is also EGFR-transactivation-dependent and this pathway is not cell type specific but generalizable for cardiovascular cells, we compared the proliferative characteristics of 247R-expressing SMCs with control cells or cells stably expressing WT receptor in the presence or absence of EGFR, Src or MMP inhibitors AG1478, PP2 or GM6001, respectively. All three inhibitors blocked the 247R-triggered hyperproliferation reducing it to the levels observed in WT or control cells, and neither inhibitor had any statistically significant effect on the basal proliferation of control or WT cells (Fig 2A and 2B). These data demonstrate that 247R-triggered hyperproliferation in SMCs is Src/MMP/EGFR-dependent. We also confirmed these findings by Western blot analysis using the same, pathway-specific inhibitors. As demonstrated in Fig 2C, 247R expression triggers basal EGFR activation compared with control or WT receptor-expressing cells (Fig 2C, lanes 7 vs 1,4). EGFR-specific inhibitor AG1478 and MMP inhibitor GM6001 inhibited 247R-triggered EGFR phosphorylation (Fig 2C lanes 7 vs 8,9) confirming that 247R-triggered cell proliferation in SMCs is MMP/EGFR-transactivation-dependent.

Src tyrosine kinase family proteins have been suggested to function as upstream mediators of EGFR transactivation, particularly in response to the agonist stimulation of GPCRs [44]. We have also demonstrated that in 247R-expressing cardiomyoblasts, Src-kinase is highly activated compared to WT receptor-expressing cells and the cell proliferation is inhibited with Src kinase-specific inhibitor PP2 [41]. Therefore, we hypothesized that Src can be involved, and play a role in 247R-mediated EGFR transactivation in SMCs. To test this hypothesis, we examined phosphorylation of EGFR at Tyr(Y)-845 site, known to be phosphorylated by Src kinase [45]. As shown in Fig 2C, Src-specific pY845-EGFR site is also upregulated in 247R-expressing cells compared to control or WT receptor-expressing cells (lane 7 vs 1,4). AG1478 and GM6001 inhibited the level of pY845-EGFR in 247R-expressing cells but not in WT cells. The fact that MMP or EGFR inhibitors did not inhibit EGFR activation in WT receptor-expressing cells, and slightly, but statistically non-significantly upregulated them, demonstrates that there is no EGFR transactivation in these cells under basal conditions. Src-specific inhibitor PP2 also resulted in statistically significant inhibition of EGFR transactivation in 247R-expressing cells, but not in control or WT cells, further confirming that 247R-triggered cell proliferation is Src-dependent (Fig 2D lane 5 vs 6). PP2 inhibited phosphorylation of Src in 247R-expressing as well as in WT cells, yet inhibition of pY-Src in WT cells did not affect proliferation in WT cells while it inhibited proliferation in 247R-expressing cells (Fig 2B). These data demonstrate, as mentioned above, that proliferation in 247R cells is Src/EGFR-dependent.

### EGFR-mediated ERK phosphorylation is responsible for induction of cell cycle progression in 247R-expressing smooth muscle cells

**Fig 3:**
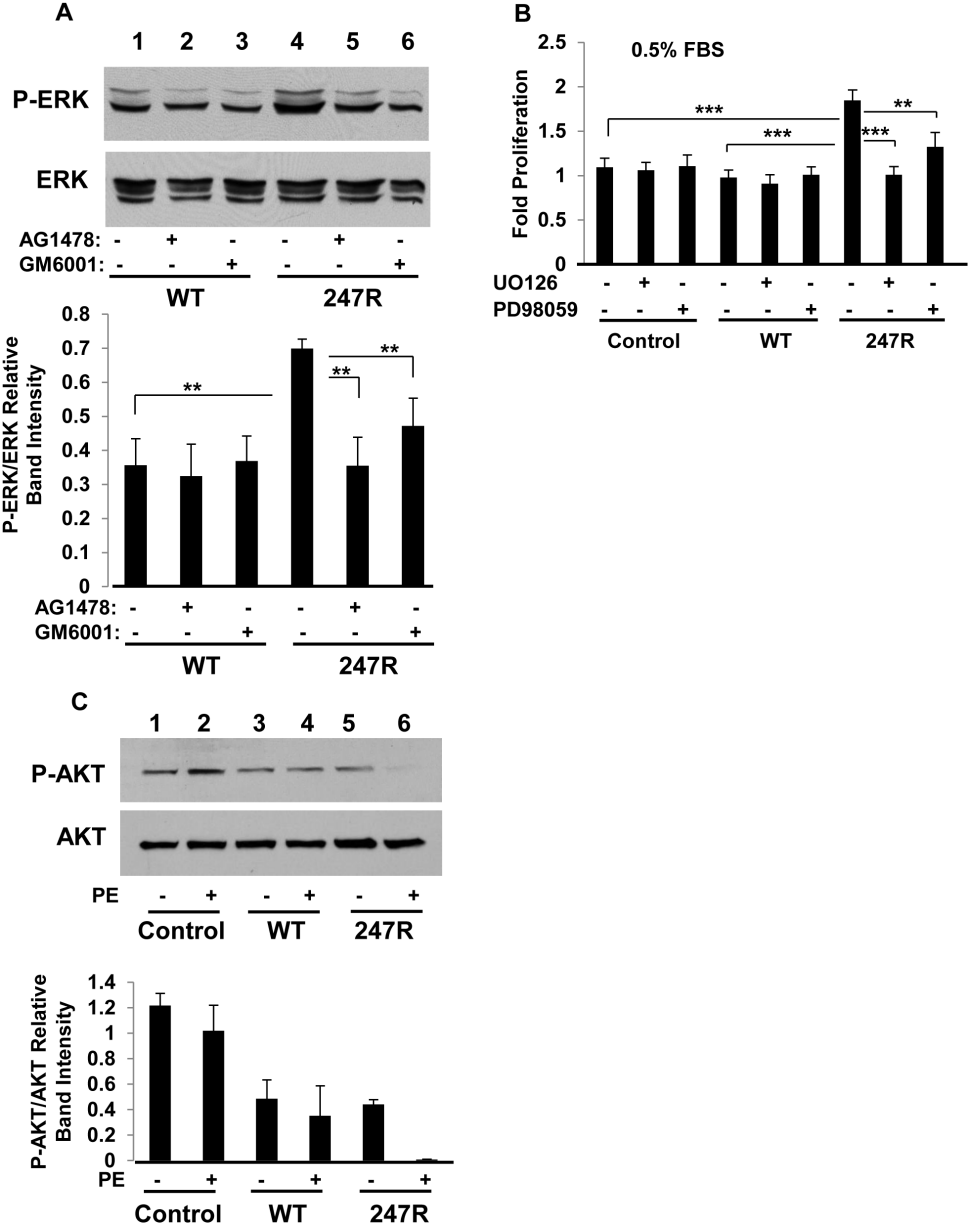
ERK is constitutively activated in 247R-expressing cells via MMP/EGFR transactivation; AKT is not activated. (A) A-10 SMCs expressing WT or 247R receptors were cultured in 0.5% FBS-containing medium in the presence or absence of AG1478 (EGFR-specific inhibitor) or GM6001 (general MMP inhibitor) or (B) either PD98059 or UO126 MEK inhibitors or (C) agonist for 48h. Cell lysates were analyzed by Western blotting and cell proliferation was evaluated by light microscopy. Lower panels depict relative intensities of phospho-protein bands as determined by densitometric analysis. Data represent mean ± SE of 5 (A), 4 (B) or 3 (C) independent experiments and analyzed by two-way ANOVA followed by post-hoc Tukey’s test.

It has been demonstrated that for some GPCRs ERK and AKT are the key intracellular molecules downstream of agonist-induced EGFR transactivation [46]. Therefore, we examined the levels of ERK and AKT phosphorylation in WT and 247R-expressing cells. To establish whether basal ERK activation in 247R cells is a result of MMP/EGFR transactivation and that it is downstream of EGFR, the cells were treated with EGFR or MMP inhibitors and cell lysates were immunoblotted with phospho-ERK antibody. Basal levels of phosphorylated ERK in 247R cells are significantly increased compared to that in WT cells (Fig 3A, lane 4 vs 1). However, when treated with inhibitors, activated phospho-ERK levels are reduced significantly, demonstrating that the increased proliferation in 247R-expressing cells is MMP/EGFR/ERK-dependent. Phospho-ERK levels in WT cells are not substantially changed (Fig 3A, lanes 2,3 vs 1). To further confirm that ERK is involved in EGFR transactivation-dependent SMC proliferation, cells were pretreated (or not) with a pharmacological inhibitor of mitogen-activated protein kinase (MEK) PD98059 or UO126 for 48h. Pretreatment with PD98059 or UO126 ablated virtually all ERK activation above basal levels indicating that ERK activation regulates MMP-2/EGFR-mediated cell proliferation in 247R-expressing cells (Fig 3B). However, these inhibitors did not affect proliferation of control or WT-expressing cells (Fig 3B). Western blot analyses of basal AKT phosphorylation (activation) shows that AKT is not activated in 247R-expressing cells, thus indicating that observed hyperproliferation in 247R cells is not AKT-dependent. In both WT and 247R cells, the AKT levels are slightly downregulated compared to control (Fig 3C, lanes 3,4,5,6 vs 1,2); PE stimulation nearly abolishes AKT phosphorylation in 247R-expressing cells (Fig 3C, lanes 5 vs 6), while it has no apparent effect in control or WT cells.

### 247R expression triggers agonist-dependent hypertrophy that is also EGFR-transactivation-dependent

**Fig 4:**
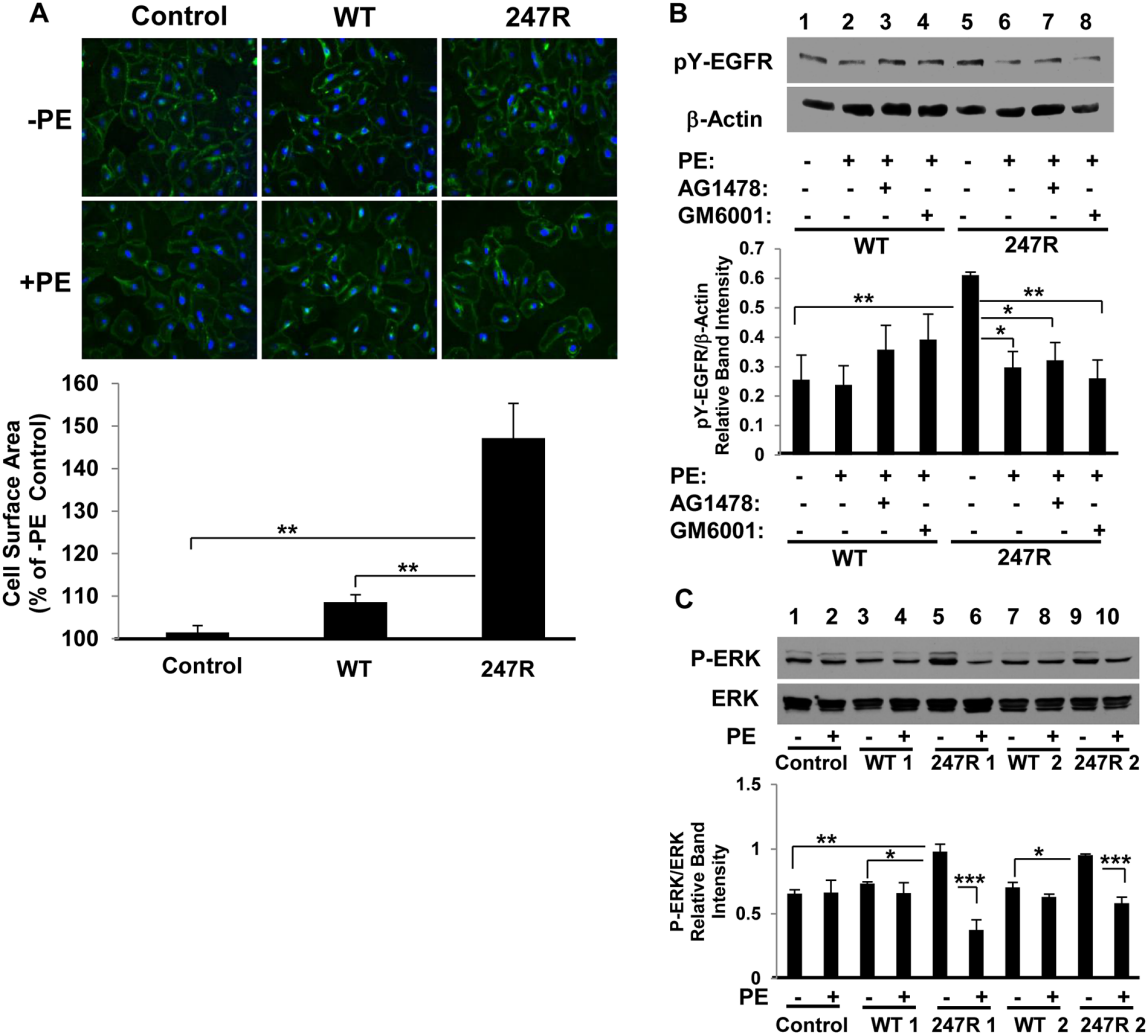
247R expression in SMCs triggers hypertrophy in response to agonist stimulation which is MMP/EGFR transactivation-dependent. (A) Control A-10 SMCs, or cells expressing WT or 247R receptors were cultured for 48h in 0.5% FBS-containing medium in the presence or absence of 10μM PE, followed by cell membrane staining with wheat germ agglutinin. Average cell surface area was evaluated by Image J software at 20x magnification. (B,C) Cells were cultured for 48h in 0.5% FBS-containing medium in the presence or absence of EGFR-specific inhibitor AG1478, MMP inhibitor GM6001 and/or 10μM PE. Cell lysates were analyzed by Western blotting. Data represent mean ± SE of 3 (A, C) or 4 (B) independent experiments and analyzed by one-way ANOVA followed by post-hoc Tukey’s test (A) or two-way ANOVA followed by post-hoc Tukey’s (B) or Bonferroni (C) tests.

Agonist (PE) treatment triggers hypertrophy in 247R-expressing cells. As demonstrated in Fig 4A, under low serum conditions, there is a robust (~50%) increase in cell size in 247R-expressing cells in response to PE stimulation compared to non-stimulated cells. Cell surface areas quantified by immunofluorescent staining of the cells with wheat germ agglutinin are: control 101.51%±1.58, WT 108.62%±1.74, and 247R 147.14%±8.16. Basal (non-stimulated) state is indicated as 100% for each cell type. Similar results were obtained in cells grown in 10% FBS-containing medium (S2 Fig).

Agonist treatment also significantly inhibited the levels of pY-EGFR in 247R cells, but not in WT (Fig 4B). When cells were cultured in the presence of EGFR or MMP inhibitors and PE, the phosphorylation of EGFR was not altered compared with PE alone (Fig 4B lanes 7,8 respectively, and densitometric analysis underneath). These data demonstrate that in SMCs both 247R-triggered constitutive hyperproliferation, as well as the observed 247R-triggered hypertrophy signaling pathways, are EGFR-dependent. Agonist treatment also inhibited ERK phosphorylation in 247R-expressing cells (Fig 4C, lanes 5 vs 6 and 9 vs 10, each shown for two independent clones), while it had no statistically significant effect on phospho-ERK levels in control (lanes 1 vs 2) or WT-expressing cells (lanes 3,4, and 7,8 vs 1,2).

### 247R-triggered hyperproliferation in SMCs is MMP-2-dependent

**Fig 5:**
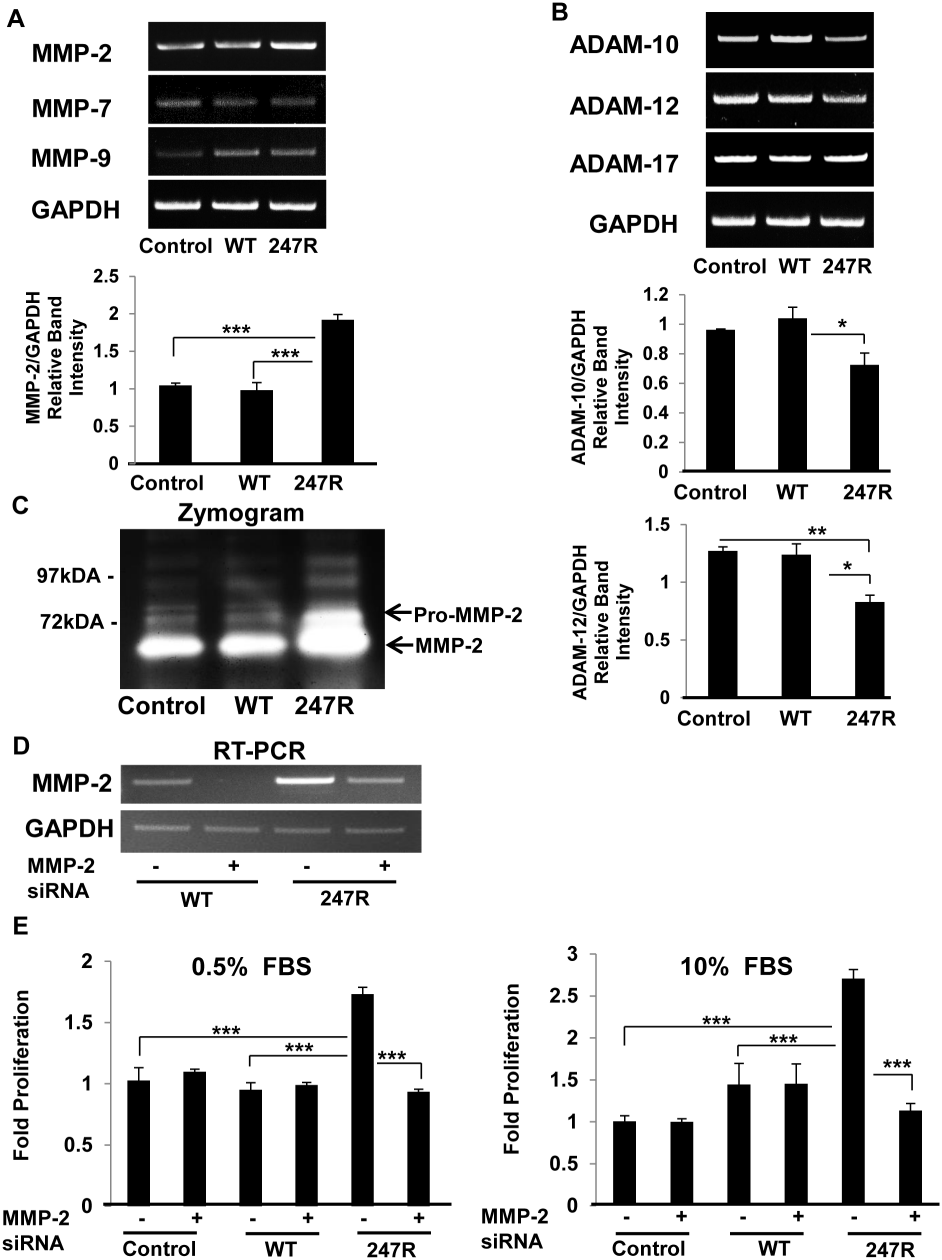
247R-triggered hyperproliferation is MMP-2-dependent in SMCs. Control A-10 cells, or cells stably expressing WT or 247R receptors were cultured in serum free medium for 24h and MMP or ADAM expression levels were analyzed by RT-PCR (A,B) or Zymogram (C). Graphs depict relative band intensities normalized to corresponding GAPDH bands as determined by densitometric analysis. Data represent mean ± SE of 3 independent experiments. (D) Cells were transfected with MMP-2-specific or non-targeting negative control siRNAs and specificity of siRNAs was determined by RT-PCR; GAPDH was used as an internal control or (E) after transfection cells were cultured in 0.5% or 10% FBS containing medium for 48h and cell proliferation was analyzed by cell counting. Data represent mean ± SE of 3 independent experiments, each performed in triplicates and analyzed by one-way (A,B) or two-way (E) ANOVA followed by post-hoc Tukey’s test.

It is known that a number of MMPs and ADAMs such as MMP-2, 9, and ADAM-10, 12, 17 are expressed in vascular SMCs [47,48]. To characterize the involvement of various MMPs in 247R signaling pathway, we evaluated the expression of MMPs and ADAMs at the RNA level in control, WT and 247R-expressing SMCs. Our data reveal significant upregulation of MMP-2 levels (also confirmed by zymography) and relatively unchanged levels for MMP-7 and MMP-9 in 247R-expressing cells compared to control or WT receptor-expressing cells (Fig 5A and 5C). Interestingly, levels of ADAM-10 and ADAM-12 were downregulated in 247R cells compared to that in control or WT cells (Fig 5B), while the levels of ADAM-17 remained unchanged.

To confirm involvement of MMP-2 in increased proliferation of 247R-expressing cells, control A-10 SMCs or cells expressing WT or 247R receptors were transfected with non-targeting negative control or MMP-2-specific siRNA and cell proliferation was evaluated. Specificity of siRNA was tested prior to proliferation assays by RT-PCR, which demonstrates that siRNAs used are specific and substantially reduce the MMP-2 mRNA levels (Fig 5D). These siRNAs were used in the proliferation assays and our results demonstrate that knockdown of MMP-2 mRNA significantly reduces cell proliferation to near normal levels in 0.5% or 10% FBS-containing medium as determined by cell counts (Fig 5E). These data demonstrate that MMP-2 is involved in increased proliferation of 247R-expressing SMCs.

### 247R-triggered hyperproliferation in SMCs is β-Arrestin1-dependent, while hypertrophy is β-Arrestin1-independent

**Fig 6:**
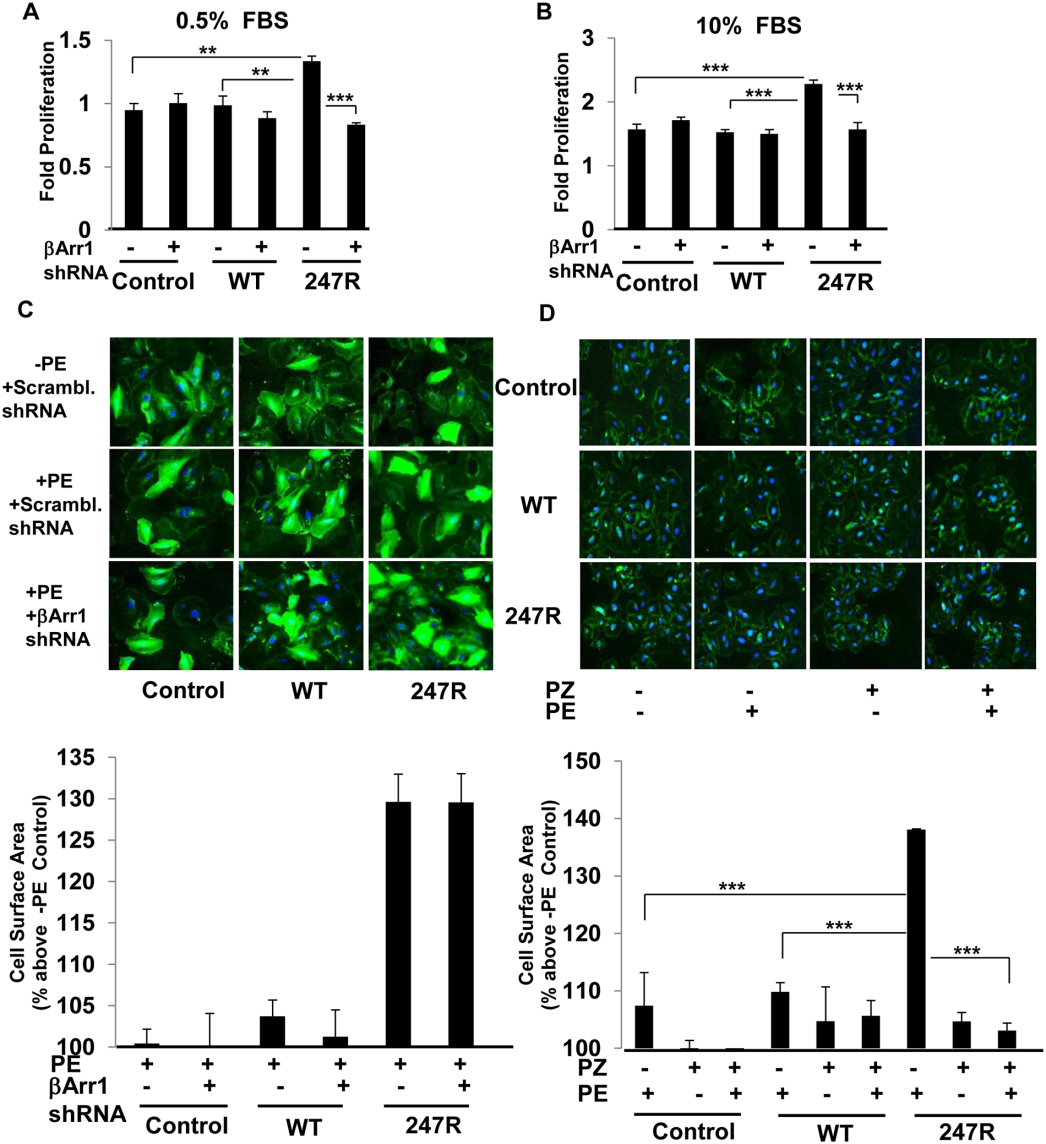
247R-triggered hyperproliferation but not hypertrophy is mediated by β-arrestin1. Control A-10 cells, or cells stably expressing WT or 247R receptors were transfected with EGFP-tagged scrambled or β-arrestin1-specific shRNAs and cultured for 24h in (A) 0.5% or (B) 10% FBS-containing medium. Cell numbers were evaluated by light microscopy. Data represent mean ± SE of 3 independent experiments, each performed in triplicates, (C) Cells were cultured for 48h in the presence or absence of PE, (D) PE or/and PZ followed by cell membrane staining with wheat germ agglutinin. Average cell surface area was evaluated by Image J software at 20x cell magnification. In (C) cell surface area was evaluated only for EGFP positive cells. Data represent mean ± SE of 3 independent experiments and analyzed by two-way ANOVA followed by post-hoc Tukey’s test.

It has been demonstrated that in cardiovascular cells GPCR-mediated EGFR transactivation could be β-arrestin-dependent [45,49]. To examine whether 247R triggers β-arrestin1-mediated signaling pathway in SMCs, we examined effects of β-arrestin1-specific shRNA on proliferation of 247R-expressing cells. β-arrestin1-specific, but not control scrambled shRNA, inhibited constitutive hyperproliferation of 247R-expressing cells cultured in 0.5% FBS-containing medium (Fig 6A). Similar results in response to treatment with β-arrestin1-specific shRNA were observed in 247R cells cultured in 10% FBS-containing medium (Fig 6B), indicating that in SMCs 247R-triggered increased cellular proliferation is β-arrestin1-dependent.

To examine whether agonist-dependent hypertrophy in 247R-expressing cells is β-arrestin1-dependent, cells were transfected with EGFP-tagged scrambled or β-arrestin1-specific shRNAs (the same shRNAs used in proliferation assays described above), and cell surface areas for only EGFP-positive cells were determined. As demonstrated in Fig 6C, PE stimulation triggered robust hypertrophy in 247R-expressing cells and β-arrestin1 knockdown did not inhibit hypertrophy. These data demonstrate that agonist triggered hypertrophy in 247R-expressing cells is not β-arrestin1-dependent. We also performed hypertrophy assays in the presence of α_1a_AR inverse agonist prazosin. Similar to stimulation with 10μM PE (Fig 4A), 247R-expressing cells stimulated with 1μM PE exhibit enhanced hypertrophy compared with untreated cells (Fig 6D). Treatment with 1μM prazosin alone does not induce hypertrophy, but completely reverses the hypertrophic effect of PE (Fig 6D), suggesting that hypertrophy in 247R cells is G_q_-dependent.

### Smooth muscle cell contractility is increased in 247R receptor-expressing cells compared to WT cells

**Fig 7:**
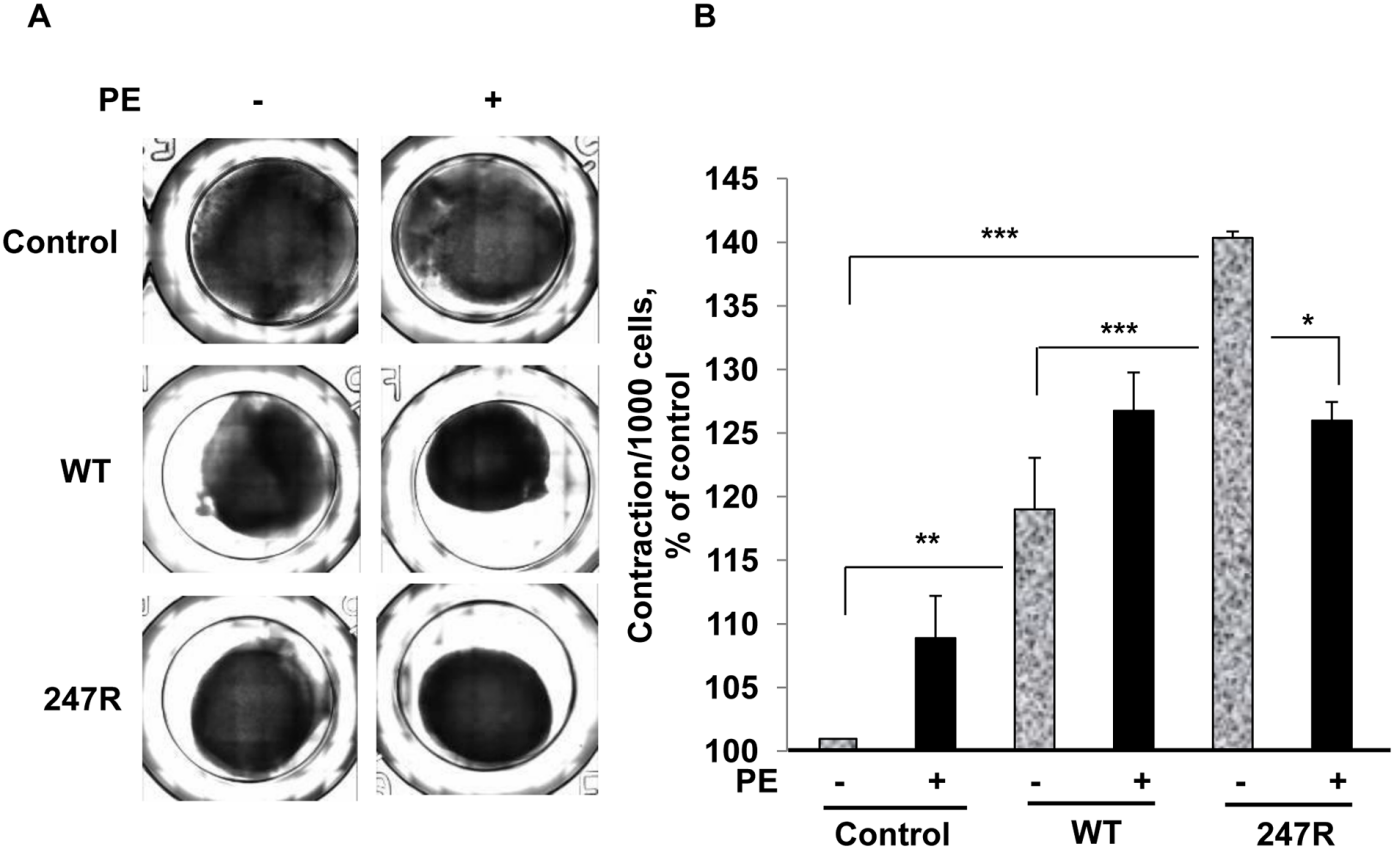
Cell contraction is increased in 247R-expressing cells compared with WT in basal (without agonist) conditions. (A) Control A-10 cells, or cells stably expressing WT or 247R receptors were plated in collagen gels with or without agonist stimulation for 4 days followed by image acquisition and evaluation of corresponding collagen gel areas. (B) Gels were digested with collagenase and cell numbers were evaluated by light microscopy. Data is presented as percent of contraction per 1000 cells above that of non-stimulated control. Data represent mean ± SE of 3 independent experiments and analyzed by two-way ANOVA followed by post-hoc Tukey’s test.

To explore whether 247R expression affects contractility of SMCs, we performed cell culture experiments in 3D collagen gels that more closely mimic physiological conditions. Control, WT and 247R-expressing SMCs were seeded in collagen gels as described in Methods and cultured in the presence or absence of agonist for 4 days. Gel contractility was evaluated by measuring the gel surface area (Fig 7A) as described [50]. Our results demonstrate that expression of WT or 247R significantly increases contraction of SMCs compared to control cells (Fig 7A). We also evaluated the cell numbers in 3D cultures and normalized to corresponding cell types. The data presented in Fig 7B reveal that contraction of 247R-expressing cells in 3D cultures is approximately two-fold higher compared to that of WT cells. These data demonstrate that in contrast to control or WT, expression of 247R genetic variant in rat SMCs results in hyperproliferation and significantly elevated contraction in 3D cultures under basal conditions. PE treatment slightly increases contraction of WT cells; however this is not statistically significant under these experimental conditions (Fig 7B). PE inhibits proliferation of 247R cells and triggers robust hypertrophy, leading to reduced contractility of 247R cells, comparable with WT receptor-expressing cells (Fig 7B).

## Discussion

In this study we demonstrate that stable expression of naturally occurring α_1a_AR-247R (247R) genetic variant, identified in a hypertensive patient, results in agonist independent hyperproliferation of vascular SMCs due to MMP-2-mediated, constitutively active coupling to the EGFR transactivation pathway that is β-arrestin1, Src-, and ERK-dependent. MMP-2 gene-specific siRNA and β-arrestin-specific shRNA inhibited 247R-triggered hyperproliferation in SMC confirming that observed hyperproliferation is MMP-2- and β-arrestin1-dependent. Further, we found that the agonist-independent hyperproliferation triggered by 247R genetic variant in SMCs is sensitive to EGFR-specific inhibitor AG1478, Src inhibitor PP2 and general MMP inhibitor GM6001. We also demonstrate that ERK, downstream of EGFR is responsible for increased hyperproliferation as confirmed by MEK kinase-specific inhibitors PD98059 and UO126. Thus, our findings reveal that 247R genetic variant-mediated hyperproliferation in SMCs is β-arrestin1/Src/MMP-2/EGFR/ERK-dependent.

**Fig 8:**
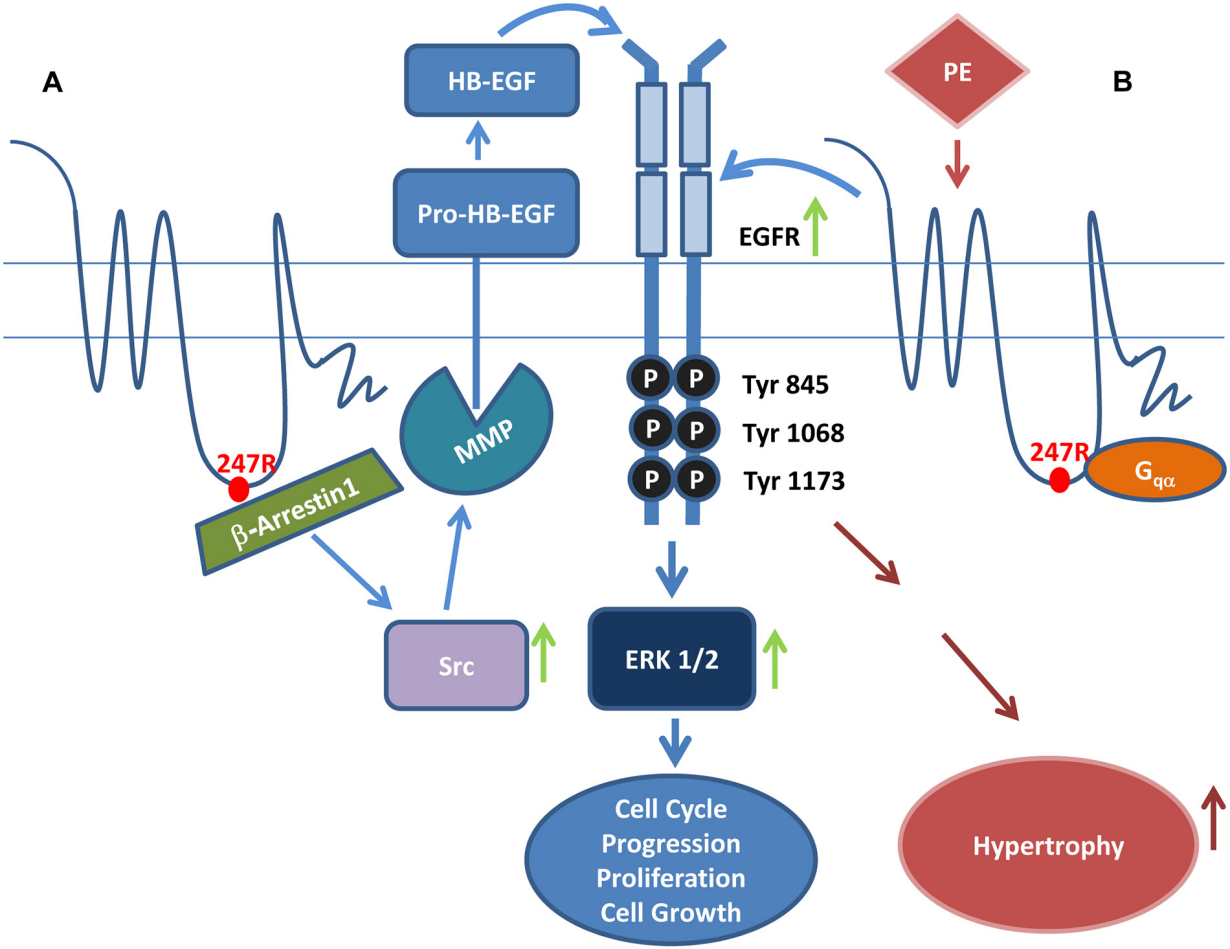
Schematic of 247R-induced constitutive hyperproliferation and agonist-dependent cell hypertrophy in SMCs. (A) 247R triggers constitutive, β-arrestin1/Src/MMP-2-dependent EGFR transactivation leading to ERK activation, cell cycle progression and cell proliferation. (B) Agonist stimulation of 247R cells triggers MMP/EGFR-dependent hypertrophy in SMCs.

Our findings also reveal that agonist stimulation of 247R-expressing cells leads to statistically significant SMC hypertrophy compared to WT cells and this hypertrophic pathway is also MMP/EGFR-dependent. Interestingly β-arrestin1-specific shRNAs did not inhibit agonist-mediated hypertrophy in 247R genetic variant expressing cells, demonstrating that hypertrophy in these cells is not β-arrestin1-dependent. Further, the competitive, α_1a_AR inverse agonist prazosin inhibited agonist-mediated hypertrophy suggesting G_q_-dependent pathway in 247R expressing cells (Fig 8).

Another novel finding in this study is that 247R-expressing cells demonstrate significant hyperproliferation in 3D cultures as well as increased contraction compared to WT. Agonist treatment leads to hypertrophy and reduces SMC contraction in 247R-expressing cells.

The mechanism of EGFR transactivation and its pathophysiological significance are currently major topics of signal transduction research. Abnormal regulation of GPCR-mediated vascular SMC proliferation and migration are the main forces inducing pathological conditions such as hypertension or atherosclerosis. In response to hemodynamic or biomechanical stress the vascular wall undergoes functional, mechanical and structural changes in hypertensive patients [51]. At the molecular level, numerous factors have been implicated in vascular remodeling of hypertension, including activation of RAS family proteins, imbalance between MMPs and tissue inhibitors of metalloproteinases (TIMPs), and aberrant G protein-coupled receptor signaling [52].

Classically α_1a_ARs couple predominantly to G_q_ proteins. Receptor-G protein signaling is terminated through β-arrestins presumably via physically interfering with receptor coupling to cognate G protein (desensitization) as well as removing receptors from the cell surface through endocytic machinery via internalization. However, recent findings reveal that β-arrestins also act as signal transducers and adaptors that scaffold a variety of signaling molecules, leading to mitogen-activated protein kinase (MAPK) activation, DNA synthesis, and cell migration [53, 31]. Different β-arrestins have been identified as signal transducers for various GPCRs and depending on the receptor and cell type, β-arrestin1 or 2 or both may be involved in signaling processes. For example, both β-arrestin1 and 2 are required for β-arrestin-dependent ERK activation mediated by β_2_-adrenergic [54] and parathyroid hormone [55] receptors, while β-arrestin1 and 2 show reciprocal functions in AT1R signaling [56]. We have also demonstrated that β-arrestin1, but not β-arrestin2 is responsible for 247R-mediated constitutive hyperproliferation in Rat-1 fibroblasts as well as in cardiomyoblasts [29, 41]. Several physiologic consequences of β-arrestin-dependent signaling have been discovered in recent years. β_1_-adrenergic receptors have been shown to mediate β-arrestin-dependent transactivation of the EGFR thus promoting activation of the cardioprotective pathway in the heart that counteracts the effects of catecholamine toxicity [32]. Here we demonstrate that 247R-triggered hyperproliferation in SMCs is β-arrestin1-dependent and could be inhibited by β-arrestin1-specific shRNAs. Our findings also demonstrate that 247R genetic variant-triggered hyperproliferation is a generalizable mechanism effective in different cardiovascular cells including fibroblasts [29], cardiomyocytes [41] and smooth muscle cells.

Transactivation of EGFR by certain GPCR agonists was originally reported by Ullrich and co-authors in Rat-1 fibroblasts [57]. This pathway is now recognized as an important mechanism for GPCR signaling that is essential for mediating GPCR functions [58]. Recent findings demonstrate that EGFR transactivation by GPCRs is facilitated by a second messenger directly and/or via signal transduction pathways operated by second messengers, such as elevation of intracellular Ca^2+^, activation of PKC and generation of ROS [20,23,59]. Cytosolic non-receptor tyrosine kinases, such as Src or PYK2, may also be involved in EGFR transactivation [60, 61].

Here we also demonstrate that Src-specific Y845 phosphorylation of EGFR [62, 63] is upregulated in 247R-expressing cells. As shown by Sato et al., Y845 phosphorylation of EGFR by Src requires not only the catalytic activity of Src but also the binding of EGFR ligands to EGFR, which triggers conformational changes of the receptor, exposing Y845 site for phosphorylation by Src kinase [64]. It has been reported that upon activation of β_2_- or α_2_-adrenergic receptors, Src-specific Y845-EGFR site could be activated by direct phosphorylation of the EGFR receptor (ligand-independent) [65, 66] or by indirect, ligand-dependent mechanisms, as recently reported [67]. The authors reveal that in A431 cells thrombospondins are capable of inducing EGFR activation (involving the phosphorylation of Y845 and other tyrosine residues) and cell migration through activation of MMP-9 (but not through direct binding to EGFR), while in vascular smooth muscle cells Src-EGFR signaling is ligand-independent [68]. We have demonstrated that in cardiomyoblasts 247R triggers Src-mediated ligand-dependent and ligand-independent EGFR transactivation [41]. In the present study we establish that Src kinase activity is upregulated in SMCs expressing 247R. 247R-mediated EGFR transactivation is Src/MMP-dependent as evidenced by upregulation of Src-specific Y845 phosphorylation as well as being sensitive to EGFR and MMP inhibitors, thus unraveling MMP- and extracellular ligand-dependent activation of EGFR. Further, Src-specific inhibitor PP2 inhibited Src activation not only in 247R cells, but also in WT receptor-expressing cells. Interestingly, reduction in Src activity inhibited only 247R-triggered hyperproliferation in SMCs but had no effect on proliferation of cells expressing WT receptor demonstrating that Src is involved in 247R-induced hyperproliferation signaling pathway.

Another mechanism for EGFR transactivation by a GPCR that was recently proposed involves second messenger-sensitive metalloprotease activation and subsequent shedding of EGFR ligand(s) from its membrane-bound inactive pro-form to its active conformation, which plays critical roles in vascular remodeling [69].

The MMPs are a family of more than 25 Zn^2+^-requiring proteases with overlapping activities against a variety of extracellular matrix components, with approximately 14 of them expressed in vascular SMCs. MMP-2 is constitutively expressed in vascular SMCs of normal arteries and was found to be linked to a number of pathological conditions, including atherosclerotic arteries and hypertension [70–72]. It has been reported that MMP-2 activity is increased in hypertensive rats [73] as well as in the plasma of hypertensive patients [74]. A number of ADAMs (disintegrin metalloproteinases) containing an MMP-like catalytic domain, such as ADAM-10, ADAM-15, and ADAM-17 are also expressed in vascular cells [75]. It was shown that agonist stimulation of platelet-activating factor receptor (PAFR) belonging to GPCR family enhanced MMP-2 production in rat aortic primary vascular SMCs via the activation of a β-arrestin-dependent ERK signaling pathway [76]. PAF-induced MMP-2 production was significantly attenuated by ERK inhibition using molecular and pharmacological inhibitors suggesting that ERK signaling pathway is important for PAF-induced MMP-2 production in vascular SMCs. Consistent with these findings, we have reported that in fibroblasts MMP-7 and ADAM-12 are important sheddases activated in 247R-expressing cells [29]. Here, we further expanded these studies and demonstrate that constitutive expression of 247R in SMCs significantly increases MMP-2 expression levels and activity, while MMP-7 levels are not changed and ADAM-10 and -12 levels are only slightly downregulated. It has been shown that MMP-7 and ADAM-12 are functionally connected in agonist-induced transcriptional events that may ultimately result in the development of hypertension and cardiovascular hypertrophy [69]. Therefore, it is plausible that expression of 247R in SMCs triggers constitutive activation of MMP-2 (similar to sustained agonist stimulation) and may also transcriptionally regulate expression of ADAM-12 or ADAM-10 representing a novel signaling mechanism in SMCs leading to hyperproliferation, hypertrophy and eventually to some forms of sympathetically-mediated hypertension. Indeed, MMP-2 knockdown by gene-specific siRNA led to inhibition of 247R-triggered hyperproliferation, suggesting that MMP-2 is one of the matrix metalloproteinases involved in increased hyperproliferation of 247R-expressing SMCs.

A number of GPCRs has been known to activate ERK through EGFR transactivation mechanisms in various cell types, including vascular SMCs [77, 78]. The signaling mechanisms underlying ERK1/2 activation are complex and may originate from the activation of classical G protein-regulated effectors, from cross-talk between GPCRs and receptor tyrosine kinases (including the EGF receptor) or from β-arrestin scaffolding directly on GPCRs [55]. Here, the authors reported that some differences in ERK activation are receptor and cell type-dependent. For example, in murine embryonic fibroblasts stably expressing PAR2 receptor, ERK1/2 activation was found to be mediated predominantly by a βarrestin-dependent mechanismIn HEK293 cells expressing AT1A receptor, βarrestin-dependent and G protein-dependent mechanisms were found to contribute almost equally to the activation of ERK1/2, while in SMCs both pathways independently activate ERK [45]. We have demonstrated [29, 41] that ERK is activated in β-arrestin1/MMP/EGFR transactivation-dependent pathway in Rat-1 fibroblasts and cardiomyoblasts. In the present study, we also demonstrate that 247R genetic variant-mediated constitutive EGFR-transactivation-dependent hyperproliferation in SMCs is ERK-dependent. ERK is significantly upregulated in 247R-expressing cells and is downstream of MMP/EGFR transactivation pathway because EGFR-specific AG1478 inhibitor and MMP inhibitor GM6001 significantly reduce ERK activation. MEK kinase specific inhibitors PD98059 and UO126 inhibited 247R-triggered hyperproliferation confirming that ERK is responsible for increased proliferation in 247R-expressing SMCs. While β-arrestin1 specific shRNAs inhibited 247R-triggered ERK-dependent hyperproliferation in fibroblasts, cardiomyoblasts and SMCs, α_1a_AR inverse agonist prazosin inhibited 247R-triggered hyperproliferation in SMCs but not in cardiomyoblasts or fibroblasts suggesting that prazosin effect is cell type specific. Future, comprehensive studies should be performed to understand the signaling mechanisms triggered by this genetic variant in SMCs.

Both ERK and AKT pathways could be activated downstream of tyrosine kinase receptors. In 247R-expressing SMCs AKT is not activated under the basal conditions or upon prolonged agonist stimulation. We have demonstrated that AKT could be acutely activated upon agonist treatment of WT-α_1a_AR in fibroblasts [30] but relationship to proliferation was not established.

A cross-talk between Ras/MAPK and PI3K/AKT pathways has been demonstrated [79]. In most cellular systems PI3K positively regulates the Ras/MAPK cascade, facilitating maximal ERK responses to physiological stimuli, whereas activated ERK, in turn, negatively controls the PI3K/AKT pathway [80, 81]. Also, in some cells, activation of Raf, MEK and/or ERK is enhanced by PI3K inhibition [82, 83]. Some experimental data demonstrate that cross-talk depends on the levels of receptors, scaffolding proteins and the concentrations of growth factors such as GAB and IRS [84]. Therefore, it is possible that in our cellular model with expression of the receptors at near physiological levels, a cross-talk between these two pathways takes place where increased ERK activity confers inhibitory effect on PI3K/AKT pathway. Some studies [85] found that AKT is involved in agonist-dependent α_1_AR-mediated hypertrophy in cardiomyocytes, but specific α_1_AR-subtype was not identified. Others demonstrate that the maintenance of adrenergic vascular tone by the MMP/EGFR pathway requires PI3K activation [22]. Also vascular contractility implicated pathways of α_1_-adrenoceptor-mediated EGFR transactivation in rat aorta is mainly dependent on activation of PI3K and partially dependent on ERK1/2 activation [24]. Yet, in our SMC model 247R receptor expression did not activate AKT under basal conditions or after prolonged treatment with agonist.

Prolonged catecholamine stimulation of α_1_ARs induces hypertrophy in cardiovascular cells and it is one of important risk factors for cardiovascular disorders [3, 86]. It has been demonstrated that EGFR transactivation by α_1_ARs is also involved in modulation of vascular tone as well as in growth of vascular SMCs and cardiomyocytes [19].

In cultured vascular SMCs, transactivation of EGFR by α_1_ARs has been implicated as a major pathway involved in catecholamine-induced vascular SMC hypertrophy. In this system, transactivation of EGFR by α_1_ARs leads to shedding of heparin binding EGF-like growth factor (HB-EGF) with subsequent activation of EGFR and mitogen-activated protein kinase [23,87].

In this study we demonstrate that while expression of 247R genetic variant in SMCs triggers agonist-independent and β-arrestin1/MMP-2/EGFR-dependent hyperproliferation, agonist treatment of the cells expressing 247R receptor induces pronounced hypertrophy which is also EGFR transactivation-dependent. Interestingly, β-arrestin1-specific shRNA which inhibited proliferation pathway did not have any effect on inhibition of agonist induced hypertrophy indicating that hypertrophic pathway is β-arrestin1-independent. However, α_1a_AR inverse agonist prazosin inhibited agonist-dependent hypertrophy suggesting G_q_-dependent pathway in 247R-expressing cells.

The role of α_1_ARs in the regulation of vascular tone and vessel wall contraction is well established. Recently it has been shown that α_1_-adrenoceptor stimulation could lead to EGFR transactivation-mediated contraction of isolated rat thoracic aortic rings [24]. α_1a_AR-induced constriction of isolated rat mesenteric artery was reported to be inhibited by EGFR inhibitors AG1478, MEK inhibitor PD153035 and MMP inhibitor GM6001 [25].

One of the goals in this study was to determine whether 247R receptor-mediated increased hyperproliferation observed in 2D cultures is also in effect in 3D culture system that more closely recapitulates physiological conditions. Our data demonstrate that the 247R-expressing SMCs exhibit increased hyperproliferation and contractility in 3D cultures. In these experiments we used A10 SMC line [88] stably expressing WT or 247R receptors in 3D cultures. Recently, it was demonstrated [89] that A10 cells are positive for neural stem cell markers together with other stem and SMC differentiation markers: smooth muscle alpha-actin, smooth muscle myosin heavy chain and calponin. It is known, and has been demonstrated [90] that neural stem cells can be differentiated to SMC that have contractile function, therefore this cell line is suitable for examining vascular contractile and proliferative phenotypes in vitro. Using 3D culture system, we also found that agonist treatment of 247R-expressing cells leads to hypertrophy and reduced contractility. Such changes may trigger extracellular matrix remodeling and increased stiffness of vessels further contributing to certain types of cardiovascular disorders in humans. It is conceivable that instead of blocking multiple GPCRs in certain vascular beds, blockers of EGFR transactivation (e.g. EGFR, MMP inhibitors) could exhibit therapeutic potential by simultaneously inhibiting pathological vasoconstriction and growth in hypertensive disorders, where vasoconstrictive agonists capable of transactivating EGFR, such as catecholamines and angiotensin II, are typically overexpressed and contribute to disease [25,91].

In conclusion, our novel findings demonstrate that agonist- and serum-independent EGFR transactivation by α_1a_AR-247R genetic variant leads to hyperproliferation in SMCs through β-arrestin1/Src/MMP-2/EGFR/ERK transactivation pathway and increased contractility. Our data also reveal that agonist treatment leads to robust hypertrophy and reduces the contractility suggesting that genetically-mediated alterations in α_1a_AR signaling pathways and cellular function may lead to vessel wall remodeling by narrowing and stiffening it and leading to some forms of sympathetically mediated hypertension. These findings also suggest that in different cardiovascular cells the same receptor could activate alternative modulators of the same signaling pathway (particularly the EGFR transactivation pathway) such as MMP-7 and ADAM-12 in fibroblasts or MMP-2 in SMCs and cardiomyoblasts. Thus, our findings across numerous cardiovascular cell types raise the novel paradigm that depending on the type of cells expressing the same receptor (or receptor variant), different target-specific inhibitors could be used to modulate aberrant hyperproliferative or hypertrophic pathways and restore the normal phenotype. Such findings are not only interesting scientifically; they have the possibility of having broad medical impact.

## Supporting Information

S1 Fig 247R expression in human coronary artery SMCs triggers constitutive hyperproliferation. Agonist treatment (A61603 or phenylephrine, PE) inhibits increased proliferation. Transiently transfected human coronary artery SMCs cultured in 0.5% FBS for 48h expressing 247R display serum-independent hyperproliferation compared with WT-expressing cells. Data represent mean ± SE of 3 independent experiments, each performed in triplicates and analyzed by unpaired Student’s t-test.
(TIF)

S2 Fig 247R-expressing A-10 SMCs show increased hypertrophy in response to agonist stimulation compared with WT-expressing cells. Cells were cultured for 48h in 10% FBS-containing medium in the presence or absence of 10μM PE, followed by cell membrane staining with wheat germ agglutinin. Average cell surface area was evaluated by Image J software at 20x magnification. Data represent mean ± SE of 3 independent experiments and analyzed by one-way ANOVA followed by post-hoc Tukey’s test.
(TIF)

## References

[1] Houston MC, Dzau VJ. Hypertension as a risk factor Vasc Med. Boston: Little Brown; 1992.

[2] Julius S. Sympathetic hyperactivity and coronary risk in hypertension. Hypertension. 1993;21: 886–893. 8505097 10.1161/01.hyp.21.6.886

[3] Chen L, Xin X, Eckhart AD, Yang N, Faber JE. Regulation of vascular smooth muscle growth by alpha 1-adrenoreceptor subtypes in vitro and in situ. J Biol Chem. 1995;270: 30980–30988. 8537355 10.1074/jbc.270.52.30980

[4] Xin X, Yang N, Eckhart AD, Faber JE. Alpha1D-adrenergic receptors and mitogen-activated protein kinase mediate increased protein synthesis by arterial smooth muscle. Mol Pharmacol. 1997;51: 764–775. 9145914 10.1124/mol.51.5.764

[5] Faber JE, Yang N, Xin X. Expression of alpha-adrenoceptor subtypes by smooth muscle cells and adventitial fibroblasts in rat aorta and in cell culture. J Pharmacol Exp Ther. 2001;298: 441–452. 11454904

[6] Teeters JC, Erami C, Zhang H, Faber JE. Systemic α_1A_-adrenoceptor antagonist inhibits neointimal growth after balloon injury of rat carotid artery. Am J Physiol Heart Circ Physiol. 2003;284: H385–H392. 12388268 10.1152/ajpheart.00658.2002

[7] Zhang H, Faber JE. Trophic effect of norepinephrine on arterial intima-media and adventitia is augmented by injury and mediated by different α_1_-adrenoceptor subtypes. Circ Res. 2001;89: 815–822. 11679412 10.1161/hh2101.098379

[8] Zhang H, Facemire CS, Banes AJ, Faber JE. Different α-adrenoceptors mediate migration of vascular smooth muscle cells and adventitial fibroblasts in vitro. Am J Physiol Heart Circ Physiol. 2002;282: H2364–H2370. 12003847 10.1152/ajpheart.00858.2001

[9] Rokosh DG, Simpson PC. Knockout of the alpha 1A/C-adrenergic receptor subtype: the alpha 1A/C is expressed in resistance arteries and is required to maintain arterial blood pressure. Proc Natl Acad Sci USA. 2002;99: 9474–9479. 12093905 10.1073/pnas.132552699 PMC123165

[10] Andersson KE, Gratzke C. Pharmacology of α1-adrenoceptor antagonists in the lower urinary tract and central nervous system. Nat Clin Pract Urol. 2007;4: 368–378. 17615548 10.1038/ncpuro0836

[11] Tanoue A, Koshimizu TA, Shibata K, Takeo S, Tsujimoto G. Insights into α_1_ adrenoceptor function in health and disease from transgenic animal studies. Trends Endocrinol Metab. 2003;14: 107–113. 12670735 10.1016/s1043-2760(03)00026-2

[12] Tanoue A, Nasa Y, Koshimizu T, Shinoura H, Oshikawa S, Kawai T, et al The alpha(1D)-adrenergic receptor directly regulates arterial blood pressure via vasoconstriction. J Clin Invest. 2002;109: 765–775. 11901185 10.1172/JCI14001 PMC150908

[13] Hosoda C, Koshimizu TA, Tanoue A, Nasa Y, Oikawa R, Tomabechi T, et al Two alpha1-adrenergic receptor subtypes regulating the vasopressor response have differential roles in blood pressure regulation. Mol Pharmacol. 2005;67: 912–922. 15598970 10.1124/mol.104.007500

[14] Chen Z, Minneman KP. Recent progress in α1-adrenergic receptor research. Acta Pharmacologica Sinica. 2005;26: 1281–1287. 16225747 10.1111/j.1745-7254.2005.00224.x

[15] Piascik MT, Hrometz SL, Edelmann SE, Guarino RD, Hadley RW, Brown RD. Immunocytochemical localization of the α_1B_adrenergic receptor and the contribution of this and the other subtypes to vascular smooth muscle contraction: analysis with selective ligands and antisense oligonucleotides. J Pharmacol Exp Ther. 1997;283: 854–868. 9353407

[16] Rudner XL, Berkowitz DE, Booth JV, Funk BL, Cozart KL, D’Amico EB, et al Subtype specific regulation of human vascular alpha(1)-adrenergic receptors by vessel bed and age. Circulation. 1999;100: 2336–2343. 10587338 10.1161/01.cir.100.23.2336

[17] Rozengurt E. Mitogenic signaling pathways induced by G protein-coupled receptors. J Cell Physiol. 2007;213: 589–602. 17786953 10.1002/jcp.21246

[18] Touyz RM. Recent advances in intracellular signaling in hypertension. Curr Opin Nephrol Hypertens. 2003;12: 165–174. 12589177 10.1097/00041552-200303000-00007

[19] Asakura M, Kitakaze M, Takashima S, Liao Y, Ishikura F, Yoshinaka T, et al Cardiac hypertrophy is inhibited by antagonism of ADAM12 processing of HB-EGF: metalloproteinase inhibitors as a new therapy. Nat Med. 2002;8: 35–40. 11786904 10.1038/nm0102-35

[20] Kalmes A, Daum G, Clowes AW. EGFR transactivation in the regulation of SMC function. Ann N Y Acad Sci. 2001;947: 42–55. 11795306 10.1111/j.1749-6632.2001.tb03929.x

[21] Nagareddy PR, MacLeod KM, McNeill JH. GPCR agonist-induced transactivation of the EGFR upregulates MLC II expression and promotes hypertension in insulin-resistant rats. Cardiovasc Res. 2010;87: 177–186. 10.1093/cvr/cvq030 20110336

[22] Nagareddy PR, Chow FL, Hao L, Wang X, Nishimura T, MacLeod KM, et al Maintenance of adrenergic vascular tone by MMP transactivation of the EGFR requires PI3K and mitochondrial ATP synthesis. Cardiovasc Res. 2009;84: 368–377. 10.1093/cvr/cvp230 19578070

[23] Zhang H, Chalothorn D, Jackson LF, Lee DC, Faber JE. Transactivation of epidermal growth factor receptor mediates catecholamine-induced growth of vascular smooth muscle. Circ Res. 2004;95: 989–997. 15486316 10.1161/01.RES.0000147962.01036.bb

[24] Ulu N, Gurdal H, Landheer SW, Duin M, Guc MO, Buikema H, et al α_1_-Adrenoceptor-mediated contraction of rat aorta is partly mediated via transactivation of the epidermal growth factor receptor. Br J Pharmacol. 2010;161: 1301–1310. 10.1111/j.1476-5381.2010.00829.x 20977469 PMC3000655

[25] Hao L, Du M, Lopez-Campistrous A, Fernandez-Patron C. Agonist-induced activation of matrix metalloproteinase-7 promotes vasoconstriction through the epidermal growth factor-receptor pathway. Circ Res. 2004;94: 68–76. 14656925 10.1161/01.RES.0000109413.57726.91

[26] Shah BH, Shah FB, Catt KJ. Role of metalloproteinase-dependent EGF receptor activation in alpha-adrenoceptor-stimulated MAP kinase phosphorylation in GT1–7 neurons. J Neurochem. 2006:96: 520–532. 16336626 10.1111/j.1471-4159.2005.03585.x

[27] Kreda SM, Sumner M, Fillo S, Ribeiro CM, Luo GX, Xie W, et al Alpha(1)-adrenergic receptors mediate LH-releasing hormone secretion through phospholipases C and A(2) in immortalized hypothalamic neurons. Endocrinology. 2001;142: 4839–4851. 11606452 10.1210/endo.142.11.8506

[28] Faber JE, Szymeczek CL, Salvi SS, Zhang H. Enhanced alpha1-adrenergic trophic activity in pulmonary artery of hypoxic pulmonary hypertensive rats. Am J Physiol Heart Circ Physiol. 2006;291: H2272–2281. 16798826 10.1152/ajpheart.00404.2006

[29] Oganesian A, Yarov-Yarovoy V, Parks WC, Schwinn DA. Constitutive coupling of a naturally occurring human alpha1a-adrenergic receptor genetic variant to EGFR transactivation pathway. Proc Natl Acad Sci U S A. 2011;108: 19796–19801. 10.1073/pnas.1116271108 22089237 PMC3241756

[30] Lei B, Schwinn DA, Morris DP. Stimulation of α1a Adrenergic Receptors Induces Cellular Proliferation or Antiproliferative Hypertrophy Dependent Solely on Agonist Concentration. PloSOne. 2013;8: e72430. 10.1371/journal.pone.0072430 PMC3749976 23991110

[31] DeWire SM, Ahn S, Lefkowitz RJ, Shenoy SK. β-arrestins and cell signaling. Annu Rev Physiol. 2007;69: 483–510. 17305471 10.1146/annurev.physiol.69.022405.154749

[32] Noma T, Lemaire A, Naga Prasad SV, Barki-Harrington L, Tilley DG, Chen J, et al β-Arrestin—mediated β1-adrenergic receptor transactivation of the EGFR confers cardioprotection. J Clin Invest. 2007;117: 2445–2458. 17786238 10.1172/JCI31901 PMC1952636

[33] Swaminath G, Xiang Y, Lee TW, Steenhuis J, Parnot C, Kobilka BK. Sequential binding of agonists to the β2 adrenoceptor: kinetic evidence for intermediate conformational states. J Biol Chem. 2004;279: 686–691. 14559905 10.1074/jbc.M310888200

[34] Wei H, Ahn S, Shenoy SK, Karnik SS, Hunyady L, Luttrell LM, et al Independent beta-arrestin 2 and G protein-mediated pathways for angiotensin II activation of extracellular signal-regulated kinases 1 and 2. Proc Natl Acad Sci USA. 2003;100: 10782–10782. 12949261 10.1073/pnas.1834556100 PMC196880

[35] Rajagopal S, Rajagopal K, Lefkowitz RJ. Teaching old receptors new tricks: biasing seven-transmembrane receptors. Nat Rev Drug Discov. 2010;9: 373–386. 10.1038/nrd3024 20431569 PMC2902265

[36] Reder NP, Tayo BO, Salako B, Ogunniyi A, Adeyemo A, Rotimi C, et al Adrenergic alpha-1 pathway is associated with hypertension among Nigerians in a pathway-focused analysis. PLoSOne. 2012;7: e37145. 10.1371/journal.pone.0037145 PMC3353888 22615923

[37] Masuo K. Adrenoceptor polymorphisms in hypertension and diabetes with obesity-update in 2014. Curr Hypertens Rev. 2014; 8 12, (Epub ahead of print). 25115695

[38] Femminella GD, Barrese V, Ferrara N, Rengo G. Tailoring therapy for heart failure: the pharmacogenomics of adrenergic receptor signaling. Pharmgenomics Pers Med. 2014;7: 267–273. 10.2147/PGPM.S49799 25276090 PMC4175026

[39] Hernández-Pacheco G, González-Hermosillo A, Murata C, Yescas P, Espínola-Zavaleta N, Martínez M, et al Arg347Cys polymorphism of α 1a-adrenergic receptor in vasovagal syncope. Case—control study in a Mexican population. Auton Neurosci. 2004;183: 66–71. 10.1016/j.autneu.2014.01.005 24548768

[40] Lei B, Morris DP, Smith MP, Svetkey LP, Newman MF, Rotter JI, et al Novel human alpha1a-adrenoceptor single nucleotide polymorphisms alter receptor pharmacology and biological function. Naunyn Schmiedebergs Arch Pharmacol. 2005;371: 229–239. 15900517 10.1007/s00210-005-1019-9 PMC2367253

[41] Kleine-Brueggeney M, Gradinaru I, Babaeva E, Schwinn DA, Oganesian A. Alpha1a-adrenoceptor genetic variant induces cardiomyoblast-to-fibroblast-like cell transition via distinct signaling pathways. Cell Signal. 2014;26: 1985–1997. 10.1016/j.cellsig.2014.05.007 24835978 PMC4305345

[42] Hori Y, Kashimoto T, Yonezawa T, Sano N, Saitoh R, Igarashi S, et al Matrix Metalloproteinase-2 stimulates collagen-I expression through phosphorylation of focal adhesion kinase in rat cardiac fibroblasts. Am J Physiol Cell Physiol. 2012;303: C947–C953. 10.1152/ajpcell.00401.2011 22914642

[43] Delgado-Olguin P, Huang Y, Li X, Christodoulou D, Seidma CE, Seidman JG, et al Epigenetic repression of cardiac progenitor gene expression by Ezh2 is required for postnatal cardiac homeostasis. Nat Genet. 2012;44: 343–347. 10.1038/ng.1068 22267199 PMC3288669

[44] Poppleton HM, Wiepz GJ, Bertics PJ, Patel TB. Modulation of the protein tyrosine kinase activity and autophosphorylation of the epidermal growth factor receptor by its juxtamembrane region. Arch Biochem Biophys. 1999;363: 227–236. 10068444 10.1006/abbi.1998.1095

[45] Kim J, Ahn S, Rajagopal K, Lefkowitz RJ. Independent β-Arrestin2 and G_q_/protein kinase Czeta pathways for ERK stimulated by angiotensin type 1A receptors in vascular smooth muscle cells converge on transactivation of the epidermal growth factor receptor. J Biol Chem. 2009;284: 11953–11962. 10.1074/jbc.M808176200 19254952 PMC2673264

[46] Kendall RT, Lee MH, Pleasant DL, Robinson K, Kuppuswamy D, McDermott PJ, et al Arrestin-dependent angiotensin AT1 receptor signaling regulates Akt and mTor-mediated protein synthesis. J Biol Chem. 2014;289: 26155–26166. 10.1074/jbc.M114.595728 25081544 PMC4176252

[47] Smiljanic K, Obradovic M, Jovanovic A, Djordjevic J, Dobutovic B, Jevremovic D, et al Thrombin stimulates VSMC proliferation through an EGFR-dependent pathway: involvement of MMP-2. Mol Cell Biochem. 2014;396: 147–160. 10.1007/s11010-014-2151-y 25047892

[48] Bakken AM, Protack CD, Roztocil E, Nicholl SM, Davies MG. Cell migration in response to the amino-terminal fragment of urokinase requires epidermal growth factor receptor activation through an ADAM-mediated mechanism. J Vasc Surg. 2009;49: 1296–1303. 10.1016/j.jvs.2008.12.026 19394555 PMC2691776

[49] Kim I, Tilley DG, Chen J, Salazar NC, Whalen EJ, Violin JD, et al β-Blockers alprenolol and carvedilol stimulate β-arrestin-mediated EGFR transactivation. Proc Nat. Acad Sci USA. 2008;105: 14555–14560. 10.1073/pnas.0804745105 18787115 PMC2567217

[50] Chiu CL, Digman MA, Gratton E. Cell matrix remodeling ability shown by image spatial correlation. J Biophys. 2013;Id532030. 10.1155/2013/532030 PMC3725897 23935614

[51] Touyz RM. Molecular and cellular mechanisms in vascular injury in hypertension: role of angiotensin II. Curr Opin Nephrol Hypertens. 2005;14: 125–131. 15687838 10.1097/00041552-200503000-00007

[52] Savoia C, Burger D, Nishigaki N, Montezano A, Touyz RM. Angiotensin II and the vascular phenotype in hypertension. Expert Rev Mol Med. 2011;13: e11 10.1017/S1462399411001815 21450123

[53] Patel PA, Tilley DG, Rockman HA. Physiologic and cardiac roles of β-arrestins. J Mol Cell Cardiol. 2009;46: 300–308. 10.1016/j.yjmcc.2008.11.015 19103204

[54] Shenoy SK, Drake MT, Nelson CD, Houtz DA, Xiao K, Madabushi S, et al β-Arrestin-dependent, G protein-independent ERK1/2 activation by the β2 adrenergic receptor. J Biol Chem. 2006;281: 1261–1273. 16280323 10.1074/jbc.M506576200

[55] Gesty-Palmer D, Chen M, Reiter E, Ahn S, Nelson CD, Wang S, et al Distinct β-arrestin-and G protein-dependent pathways for parathyroid hormone receptor-stimulated ERK1/2 activation. J Biol Chem 2006;281: 10856–10864. 16492667 10.1074/jbc.M513380200

[56] Ahn S, Wei H, Garrison TR, Lefkowitz RJ. Reciprocal regulation of angiotensin receptor-activated extracellular signal-regulated kinases by β-arrestins 1 and 2. J Biol Chem. 2004;279: 7807–7811. 14711824 10.1074/jbc.C300443200

[57] Daub H, Weiss FU, Wallasch C, Ullrich A. Role of transactivation of the EGF receptor in signalling by G-protein-coupled receptors. Nature. 1996;379: 557–560. 8596637 10.1038/379557a0

[58] Pierce KL, Luttrell LM, Lefkowiz RJ. New mechanisms in heptahelical receptor signaling to mitogen activated protein kinase cascades. Oncogene. 2001;20: 1532–1539. 11313899 10.1038/sj.onc.1204184

[59] Eguchi S, Numaguchi K, Iwasaki H, Matsumoto T, Yamakawa T, Utsunomiya H, et al Calcium-dependent epidermal growth factor receptor transactivation mediates the angiotensin II-induced mitogen-activated protein kinase activation in vascular smooth muscle cells. J Biol Chem. 1998;273: 8890–8896. 9535870 10.1074/jbc.273.15.8890

[60] Andreev J, Galisteo ML, Kranenburg O, Logan SK, Chiu ES, Okigaki M, et al Src and Pyk2 mediate G-protein-coupled receptor activation of epidermal growth factor receptor (EGFR) but are not required for coupling to the mitogen-activated protein (MAP) kinase signaling cascade. J Biol Chem. 2001;276: 20130–20135. 11274221 10.1074/jbc.M102307200

[61] Block ER, Tolino MA, Klarlund JK. Pyk2 activation triggers epidermal growth factor receptor signaling and cell motility after wounding sheets of epithelial cells. J Biol Chem. 2010;285: 13372–13379. 10.1074/jbc.M109.083089 20215112 PMC2859496

[62] Sato K, Sato A, Aoto M, Fukami Y. c-Src phosphorylates epidermal growth factor receptor on tyrosine 845. Biochem Biophys Res Commun. 1995;215: 1078–1087. 7488034 10.1006/bbrc.1995.2574

[63] Belsches AP, Haskell MD, Parsons SJ. Role of c-Src tyrosine kinase in EGF-induced mitogenesis. Front Biosci. 1997;2: d501–d518. 9331427 10.2741/a208

[64] Sato KI. Cellular functions regulated by phosphorylation of EGFR on Tyr845. Int J Mol Sci. 2013;14: 10761–10790. 10.3390/ijms140610761 23702846 PMC3709701

[65] Drube S, Stirnweiss J, Valkova C, Liebmann C. Ligand-independent and EGF receptor-supported transactivation: Lessons from β2-adrenergic receptor signalling. Cell Signal. 2006;18: 1633–1646. 16495036 10.1016/j.cellsig.2006.01.003

[66] Du T, Li B, Liu S, Zang P, Prevot V, Hertz L, et al ERK phosphorylation in intact, adult brain by α2-adrenergic transactivation of EGF receptors. Neurochem Int. 2009;55: 593–600. 19501623 10.1016/j.neuint.2009.05.016

[67] Liu A, Garg P, Yang S, Gong P, Pallero MA, Annis DS, et al Epidermal growth factor-like repeats of thrombospondins activate phospholipase Cγ and increase epithelial cell migration through indirect epidermal growth factor receptor activation. J Biol Chem. 2009;284: 6389–6402. 10.1074/jbc.M809198200 19129184 PMC2649082

[68] Liu YT, Song L, Templeton DM. Heparin suppresses lipid raft-mediated signaling and ligand-independent EGF receptor activation. J Cell Physiol. 2007;211: 205–212. 17226785 10.1002/jcp.20924

[69] Wang X, Chow FL, Oka T, Hao L, Lopez-Campistrous A, Kelly S, et al Matrix metalloproteinase-7 and ADAM-12 (a disintegrin and metalloproteinase-12) define a signaling axis in agonist-induced hypertension and cardiac hypertrophy. Circulation. 2009;119: 2480–2489. 10.1161/CIRCULATIONAHA.108.835488 19398663

[70] Newby AC. Matrix metalloproteinases regulate migration, proliferation, and death of vascular smooth muscle cells by degrading matrix and non-matrix substrates. Cardiovasc Res. 2006;69: 614–624. 16266693 10.1016/j.cardiores.2005.08.002

[71] Lee SJ, Seo KW, Yun MR, Bae SS, Lee WS, Hong KW, et al 4-Hydroxynonenal enhances MMP-2 production in vascular smooth muscle cells via mitochondrial ROS-mediated activation of the Akt/NF-kappaB signaling pathways. Free Radic Biol Med. 2008;45: 1487–1492. 18805481 10.1016/j.freeradbiomed.2008.08.022

[72] Risinger GM Jr, Hunt TS, Updike DL, Bullen EC, Howard EW. Matrix metalloproteinase-2 expression by vascular smooth muscle cells is mediated by both stimulatory and inhibitory signals in response to growth factors. J Biol Chem. 2006;281: 25915–25925. 16854986 10.1074/jbc.M513513200

[73] Watts SW, Rondelli C, Thakali K, Li X, Uhal B, Pervaiz MH, et al Morphological and biochemical characterization of remodeling in aorta and vena cava of DOCA-salt hypertensive rats. Am J Physiol Heart Circ Physiol. 2007;292: H2438–H2448. 17237246 10.1152/ajpheart.00900.2006

[74] Derosa G, D'Angelo A, Ciccarelli L, Piccinni MN, Pricolo F, Salvadeo S, et al Matrix metalloproteinase-2, -9, and tissue inhibitor of metalloproteinase-1 in patients with hypertension. Endothelium. 2006;13: 227–231. 16840178 10.1080/10623320600780942

[75] Lemarié CA, Tharaux PL, Lehoux S. Extracellular matrix alterations in hypertensive vascular remodeling. J Mol Cell Cardiol. 2010;48: 433–439. 10.1016/j.yjmcc.2009.09.018 19837080

[76] Kim YH, Lee SJ, Seo KW, Bae JU, Park SY, Kim EK, et al PAF enhances MMP-2 production in rat aortic VSMCs via a β-arrestin2-dependent ERK signaling pathway. J Lipid Res. 2013;54: 2678–2686. 10.1194/jlr.M037176 23911909 PMC3770081

[77] Shah BH, Yesilkaya A, Olivares-Reyes JA, Chen HD, Hunyady L, Catt KJ. Differential pathways of angiotensin II-induced extracellularly regulated kinase 1/2 phosphorylation in specific cell types: role of heparin-binding epidermal growth factor. Mol Endocrinol. 2004;18: 2035–2048. 15143154 10.1210/me.2003-0476

[78] Eguchi S, Iwasaki H, Ueno H, Frank GD, Motley ED, Eguchi K, et al Intracellular Signaling of Angiotensin II-induced p70 S6 Kinase Phosphorylation at Ser 411 in Vascular Smooth Muscle Cells. Possible requirement of Epidermal Growth Factor Receptor, Ras, Extracellular Signal-Regulated Kinase, and Akt. J Biol Chem. 1999;274: 36843–36851. 10601235 10.1074/jbc.274.52.36843

[79] Aksamitiene E, Kiyatkin A, Kholodenko BN. Cross-talk between mitogenic Ras/MAPK and survival PI3K/Akt pathways: a fine balance. Biochem Soc Trans. 2012;40: 139–146. 10.1042/BST20110609 22260680

[80] Kiyatkin A, Aksamitiene E, Markevich NI, Borisov NM, Hoek JB, Kholodenko BN. Scaffolding protein Grb2-associated binder 1 sustains epidermal growth factor-induced mitogenic and survival signaling by multiple positive feedback loops. J Biol Chem. 2006;28: 19925–19938. 10.1074/jbc.M600482200 PMC2312093 16687399

[81] Hayashi H, Tsuchiya Y, Nakayama K, Satoh T, Nishida E. Down-regulation of the PI3-kinase/Akt pathway by ERK MAP kinase in growth factor signaling. Genes Cells. 2008;13: 941–947. 10.1111/j.1365-2443.2008.01218.x 18691227

[82] Choi WS, Sung CK. Inhibition of phosphatidylinositol-3-kinase enhances insulin stimulation of insulin receptor substrate 1 tyrosine phosphorylation and extracellular signal-regulated kinases in mouse R- fibroblasts. J Recept Signal Transduction Res. 2004;24: 67–83. 10.1081/rrs-120034229 15344880

[83] Serra V, Scaltriti M, Prudkin L, Eichhorn PJ, Ibrahim YH, Chandarlapaty S, et al PI3K inhibition results in enhanced HER signaling and acquired ERK dependency in HER2-overexpressing breast cancer. Oncogene. 2011;30: 2547–2557. 10.1038/onc.2010.626 21278786 PMC3107390

[84] Borisov N, Aksamitiene E, Kiyatkin A, Legewie S, Berkhout J, Maiwald T, et al Systems-level interactions between insulin—EGF networks amplify mitogenic signaling. Mol Syst Biol. 2009; 5: 256 10.1038/msb.2009.19 19357636 PMC2683723

[85] Guo J, Gertsberg Z, Ozgen N, Steinberg SF. p66Shc Links 1-Adrenergic Receptors to a Reactive Oxygen Species—Dependent AKT-FOXO3A Phosphorylation Pathway in Cardiomyocytes. Circ Res. 2009;104: 660–669. 10.1161/CIRCRESAHA.108.186288 19168439 PMC2861587

[86] Barki-Harrington L, Perrino C, Rockman HA. Network integration of the adrenergic system in cardiac hypertrophy. Cardiovasc Res. 2004; 63: 391–402. 15276464 10.1016/j.cardiores.2004.03.011

[87] Li Y, Zhang H, Liao W, Song Y, Ma X, Chen C, et al Transactivated EGFR mediates α1-AR-induced STAT3 activation and cardiac hypertrophy. Am J Physiol-Heart C. 2011;301: H1941–H1951. 10.1152/ajpheart.00338.2011 21856923

[88] Rao RS, Miano JM, Olson EN, Seidel CL. The A10 cell line: a model for neonatal, neointimal, or differentiated vascular smooth muscle cells. Cardiovasc Res. 1997;36: 118–126. 9415280 10.1016/s0008-6363(97)00156-9

[89] Kennedy E, Hakimjavadi R, Greene C, Mooney CJ, Fitzpatrick E, Collins LE, et al Embryonic rat vascular smooth muscle cells revisited-a model for neonatal, neointimal SMC or differentiated vascular stem cells. Vasc Cell. 2014;6: 6 10.1186/2045-824X-6-6 24628920 PMC3995523

[90] Oishi K, Ogawa Y, Gamoh S, Uchida MK. Contractile responses of smooth muscle cells differentiated from rat neural stem cells. Journal of Physiology. 2002:540: 139–152. 11927676 10.1113/jphysiol.2001.013278 PMC2290205

[91] Galis ZS, Khatri JJ. Matrix metalloproteinases in vascular remodeling and atherogenesis: the good, the bad, and the ugly. Circ Res. 2002;90: 251–262. 11861412

