# Supplementary figures and images for: Alpha1a-Adrenoceptor Genetic Variant Triggers Vascular Smooth Muscle Cell Hyperproliferation and Agonist Induced Hypertrophy via EGFR Transactivation Pathway
Source: PLoS One. 2015 Nov 16;10(11):e0142787. doi: 10.1371/journal.pone.0142787 (PMC4646490; doi:10.1371/journal.pone.0142787)

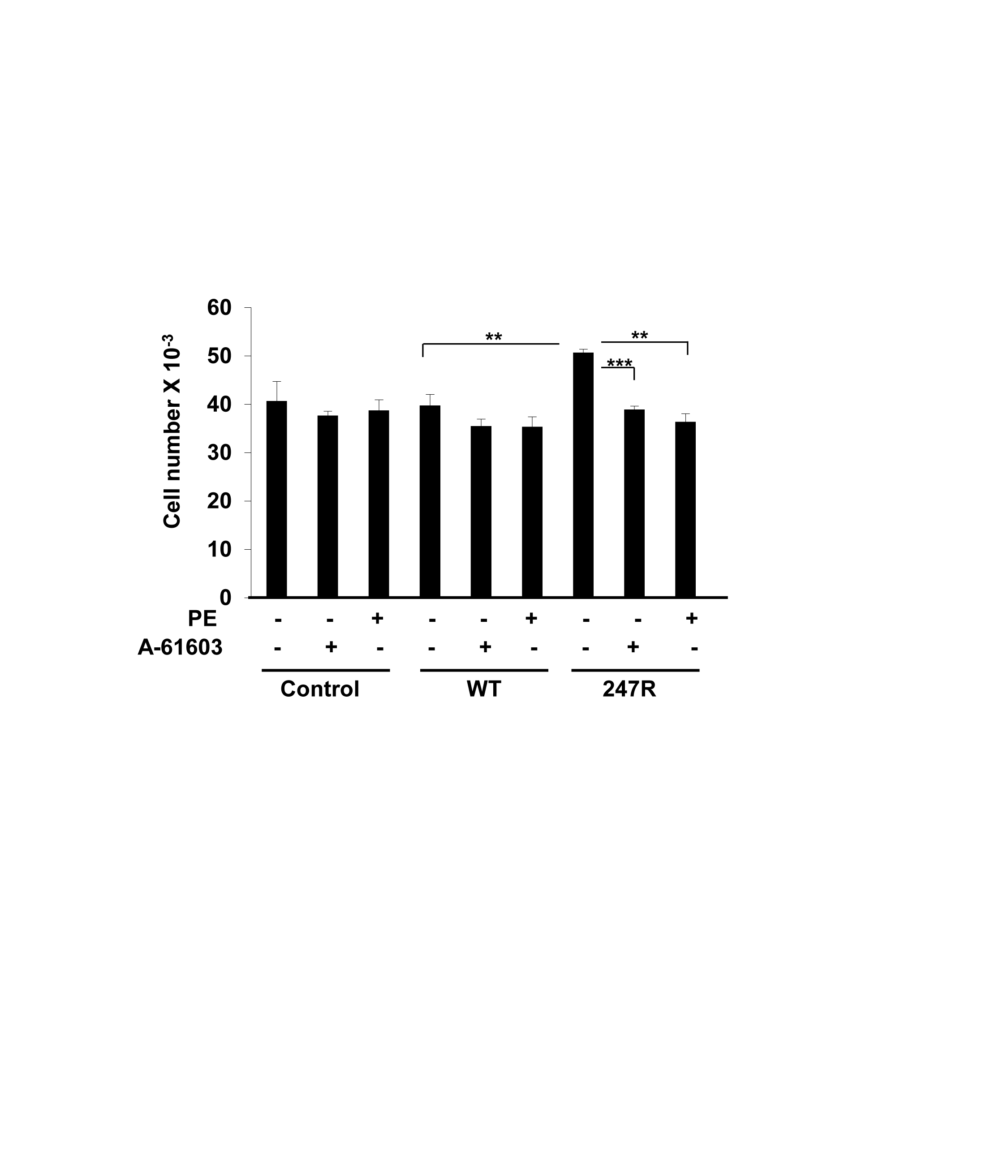

Supplement: S1 Fig — Transiently transfected human coronary artery SMCs cultured in 0.5% FBS for 48h expressing 247R display serum-independent hyperproliferation compared with WT-expressing cells. Data represent mean ± SE of 3 independent experiments, each performed in triplicates and analyzed by unpaired Student’s t-test. (TIF) [file pone.0142787.s001.tif]

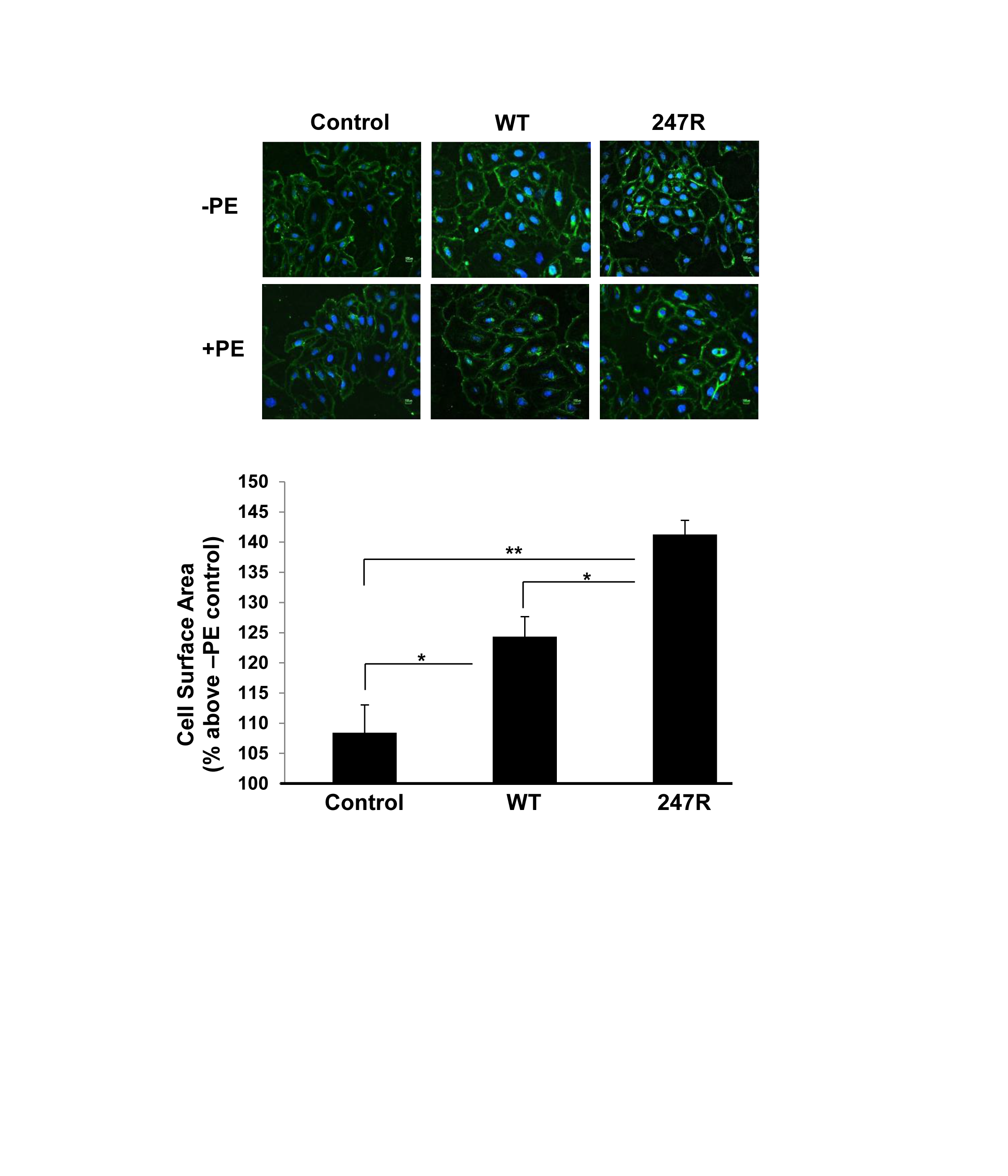

Supplement: S2 Fig — Cells were cultured for 48h in 10% FBS-containing medium in the presence or absence of 10μM PE, followed by cell membrane staining with wheat germ agglutinin. Average cell surface area was evaluated by Image J software at 20x magnification. Data represent mean ± SE of 3 independent experiments and analyzed by one-way ANOVA followed by post-hoc Tukey’s test. (TIF) [file pone.0142787.s002.tif]
